# Effects of Telehealth Interventions for People With Parkinson Disease: Systematic Review and Meta-Analysis of Randomized Controlled Trials

**DOI:** 10.2196/70994

**Published:** 2026-01-28

**Authors:** Minyue Sun, Fuyou Tang, Luo min, Shiyu Wen, Shuang Wang, Huiping Jiang

**Affiliations:** 1 Department of Neurology Mianyang Central Hospital, School of Medicine University of Electronic Science and Technology of China Mianyang China; 2 Mianyang Key Laboratory of Anesthesia and Neuroregulation, Department of Anesthesiology, Mianyang Central Hospital, School of Medicine University of Electronic Science and Technology of China Mianyang, Sichuan China; 3 Department of Neurology, Mianyang Central Hospital, School of Medicine University of Electronic Science and Technology of China Mianyang, Sichuan China

**Keywords:** depression, meta-analysis, motor symptoms, Parkinson disease, quality of life, systematic review, telehealth intervention

## Abstract

**Background:**

The global integration of telehealth into the management of Parkinson disease (PD) addresses critical gaps in health care access, especially for patients with limited mobility in underserved regions. Despite accelerated adoption during the COVID-19 pandemic, evidence regarding telehealth’s multidimensional efficacy remains inconsistent. Previous meta-analyses reported conflicting outcomes for quality of life (QOL), motor symptoms, and neuropsychiatric comorbidities.

**Objective:**

This study aimed to quantitatively synthesize the effects of telehealth interventions across six core PD domains: (1) QOL, (2) depression, (3) anxiety, (4) motor symptoms, (5) activities of daily living (ADL), and (6) cognition.

**Methods:**

PubMed, Embase, Cochrane Library, Scopus, and Web of Science were systematically searched until June 21, 2024. In adherence to PRISMA (Preferred Reporting Items for Systematic Reviews and Meta-Analyses) guidelines, English-language randomized controlled trials evaluating telehealth interventions for PD were included. Study quality was assessed using the Cochrane Risk of Bias tool. A dual analytical approach using random-effects models was applied to address heterogeneity. Studies reporting a single effect size were analyzed using the Hartung-Knapp-Sidik-Jonkman correction. Studies with multiple dependent effect sizes were analyzed using a 3-level random-effects meta-analysis with t-distribution inference, accounting for sampling, within-study, and between-study variance. Effect sizes were expressed as standardized mean differences (SMD) with 95% CIs. Heterogeneity was quantified using the τ^2^; prediction intervals were not calculated due to the limited number of studies. Prespecified subgroup analyses examined intervention types (digital vs traditional telehealth) and follow-up durations. Sensitivity analyses and assessments for small-study effects (multilevel Egger tests, funnel plots) were conducted.

**Results:**

A total of 15 randomized controlled trials (765 participants) demonstrated significant telehealth benefits: QOL significantly improved on the Medical Outcomes Study 36-Item Short Form Health Survey and Brunnsviken Brief Quality of Life Scale (SMD 0.39, 95% CI 0.06-0.72; *P*=.03), with marginal improvement on the Parkinson Disease Questionnaire-8 (SMD –0.42, 95% CI –0.88 to 0.03; *P*=.07). Telephone-based interventions outperformed digital approaches (*P*=.002). Depression symptoms were significantly reduced (SMD –0.64, 95% CI –0.93 to 0.34; *P*<.001), particularly with traditional telehealth (*P*<.001). Anxiety also decreased significantly (SMD –0.64, 95% CI –0.92 to 0.35; *P*=.003) with negligible heterogeneity (*I*^2^=0%). Motor symptoms improved (SMD –0.46, 95% CI –0.69 to 0.24; *P*=.001), and ADL showed substantial impairment reduction (SMD –0.79, 95% CI –1.04 to –0.54; *P*=.002). Cognition was significantly enhanced (SMD 1.12, 95% CI 0.03 to 2.20; *P*=.045) though with moderate heterogeneity (*I*^2^=52.3%) and significant publication bias (*P*<.001). Follow-up duration did not significantly moderate effects.

**Conclusions:**

Telehealth interventions significantly enhance multiple PD domains, with traditional (telephone/tablet-based) approaches demonstrating particular advantages for QOL and depression. Digital interventions showed more limited efficacy. These findings support telehealth as a multifaceted management tool for PD, although cognition outcomes require further investigation.

**Trial Registration:**

PROSPERO CRD42024520169; https://www.crd.york.ac.uk/PROSPERO/view/CRD42024520169

## Introduction

Parkinson disease (PD), currently the second-most common neurodegenerative disorder after Alzheimer disease, affects approximately 1% of individuals aged 55 years or older, typically manifesting around the age of 60 years [1]. The incidence of PD increases with advancing age, particularly among individuals older than 60 years [1]. Currently, PD affects nearly 6 million people globally [2]. By 2030, China is expected to have approximately 4.94 million patients with PD, representing nearly half of global cases [3]. This demographic shift could significantly strain national economies and health care systems [3].

Patients with PD experience a range of motor and nonmotor symptoms. Motor symptoms include tremors, rigidity, bradykinesia, postural instability, and gait freezing [4]. Nonmotor symptoms encompass cognitive decline, pain, fatigue, psychiatric conditions such as depression and anxiety, and sleep disturbances [5,6]. These symptoms substantially reduce patients’ quality of life (QOL) and increase psychological stress and physical demands on caregivers [7]. Traditionally, management and evaluation for patients with PD require visits to outpatient clinics or hospitals for advanced diagnostics and assessments [8]. However, many patients encounter significant barriers to accessing such facilities, including limited mobility, fear of falling, depressive symptoms, fatigue, and time constraints. These barriers can worsen their symptoms, delay treatment, lead to potentially life-threatening complications, and increase overall disease burden [9]. Consequently, a growing number of researchers advocate telehealth as a means to improve PD diagnosis, treatment, and rehabilitation. Telehealth aims to overcome challenges associated with in-person consultations and geographical disparities in health care resources, thereby reducing delays in treatment, decreasing morbidity and mortality, and improving QOL among patients with PD [10].

Telehealth uses digital information and communication technologies to connect patients with health care providers and deliver medical services [11]. These technologies include internet-connected desktop computers, tablets, smartphones, and wearable devices [12,13]. The COVID-19 pandemic significantly accelerated the adoption of telehealth among patients with PD, improving health care accessibility. Research supports the practicality of telehealth and underscores its perceived effectiveness by patients with PD and neurologists [10,14-16]. Neurologists can independently conduct comprehensive assessments using the Movement Disorders Society-Unified Parkinson’s Disease Rating Scale Part III (MDS-UPDRS-III), enabling more accurate evaluations in patients’ usual settings rather than clinical environments. This method provides a more accurate reflection of the patients’ actual condition [10,17-19]. Advantages of telehealth, such as time and cost efficiency, have resulted in high patient satisfaction [14-16,20-22]. Additionally, telehealth facilitates virtual monitoring for rehabilitation, psychotherapy, and advanced PD treatments [23-25]. It promotes interdisciplinary collaboration and provides education and training opportunities for physicians and health care workers in developing regions, overcoming geographic, travel, and financial barriers [10].

Numerous studies have confirmed the practicality and effectiveness of telehealth in managing patients with PD; yet, findings regarding QOL, anxiety, depression, motor function, activities of daily living (ADL), and cognitive function have been variable [8,26,27]. A meta-analysis by Chen et al [26] in 2020 demonstrated that telehealth interventions effectively reduced motor symptoms compared with traditional care. However, these interventions showed no substantial improvements in QOL, depression, cognitive functions, or balance abilities, contrasting with more recent findings [28-31]. A systematic review by Leon-Salas et al [8] published in 2023 indicated limited and inconclusive data regarding telehealth services for patients with PD. Following the COVID-19 pandemic, an influx of new studies necessitates an updated synthesis and analysis to clarify the effects of telehealth interventions on patients with PD. Additionally, a 2024 systematic review by Federico et al [27] reported telerehabilitation outcomes comparable to in-person therapy for patients with PD. However, the review focused solely on telerehabilitation for various neurological disorders without extensively evaluating broader telehealth effects in patients with PD.

Thus, the objective of this research was to systematically compile and analyze the most recent data on telehealth intervention effects in patients with PD.

## Methods

### Overview

This systematic review and meta-analysis was conducted in strict accordance with PRISMA (Preferred Reporting Items for Systematic Reviews and Meta-Analyses) guidelines (Multimedia Appendix 1) [32]. To ensure transparency and reproducibility, the study protocol was registered with PROSPERO (registration number CRD42024520169).

### Inclusion and Exclusion Criteria

The selection criteria followed the PICOS framework: (1) Population: individuals officially diagnosed with PD; (2) Interventions: telehealth or telemedicine interventions delivered via telephone, internet, or other digital communication technologies [11]; (3) Comparison/Control: studies with an experimental group compared to control groups using standard care, routine care, conventional care, or waitlist control; (4) Outcomes: assessment of intervention effects on overall health or specific behavioral and psychological symptoms associated with Parkinsonism; (5) Study type: only randomized controlled trials (RCTs).

The exclusion criteria were as follows: (1) studies published in non-English languages, (2) incomplete studies, such as research protocols or ongoing studies, (3) studies where interventions were exclusively telehealth-based without a comparison group, or where no telehealth intervention was applied, (4) studies lacking sufficient details on relevant outcome measures, and (5) studies without adequate statistical data for analysis. No publication date limitations were applied.

### Search Strategy

Two researchers (MS and FT) systematically searched 5 English-language databases (PubMed, Embase, Cochrane Library, Scopus, and Web of Science) from database inception until June 21, 2024. The comprehensive search strategy combined subject headings and keywords related to three main topics: (1) PD, (2) telehealth, and (3) RCTs. There were no limits on publication status or dates. The detailed search methods are provided in Multimedia Appendix 2. Additionally, references of included studies were manually reviewed.

### Study Selection and Data Extraction

The reference management tool EndNote X9 (Clarivate Analytics) was used for data management. After removing duplicates, 2 reviewers (MS and FT) independently screened titles and abstracts based on predefined inclusion and exclusion criteria. Potentially relevant articles underwent full-text assessment to determine eligibility. Any discrepancies were resolved through discussion, and when consensus was unattainable, a third reviewer (Luomin) was consulted for a final decision.

Data extraction used a specifically designed form. Extracted information included authors’ names, publication dates, country, study design, sample size, demographic and clinical characteristics (average age, gender distribution, and disease attributes), type and duration of interventions and control groups, outcome measures, and key study findings.

### Risk of Bias

Two researchers (MS and FT) independently assessed the methodological quality and potential bias of included studies using the Cochrane Collaboration’s Risk of Bias tool [33]. Disagreements were resolved through discussion with a third reviewer (Luomin) to achieve consensus. The tool systematically evaluates several dimensions of bias, including random sequence generation, allocation concealment, blinding of participants and personnel, handling of incomplete outcome data, and selective outcome reporting. Each study was assessed for potential selection, performance, detection, attrition, and reporting biases. Risks in each domain were categorized as low (unlikely to significantly affect results), high (likely to significantly undermine confidence), or unclear.

### Statistical Analysis

All meta-analyses were conducted using random-effects models, based on the conceptual assumption that true effects vary across studies due to inherent differences in populations, interventions, and settings, rather than on statistical metrics such as *I*^2^. A dual analytical approach incorporating the Hartung-Knapp-Sidik-Jonkman (HKSJ) adjustment was used to obtain more accurate and conservative interval estimates: for studies providing a single effect size, the HKSJ method was used to calculate 95% CIs; for studies with multiple dependent effect sizes, a 3-level random-effects meta-analysis was performed in R software using the *metafor* package (version 4.5.1; R Foundation for Statistical Computing) using t-distribution–based inference. This model accounts for sampling variance (level 1), within-study variance (level 2), and between-study variance (level 3), with t-distribution inference being analogous to the HKSJ correction for multilevel models. Heterogeneity was quantified with the τ^2^ statistic. Although 95% prediction intervals were initially planned to illustrate the expected range of true effects in similar future studies, they were not calculated due to the limited number of studies for each outcome (all *k*<10), as such intervals are unreliable with small samples. The *I*^2^ statistic is reported for descriptive purposes only, acknowledging its limited pragmatic use in conveying the magnitude of true effect variation across settings. Prespecified subgroup analyses examined intervention type (digital vs traditional telehealth) and follow-up duration. Sensitivity analyses were conducted using leave-one-out elimination. Small-study effects (for which publication bias is one possible explanation) were assessed with funnel plots and multilevel Egger tests. Statistical significance was set at 2-tailed *P*<.05. Complete analysis code is provided in Multimedia Appendix 3.

## Results

### Study Selection

**Figure 1:**
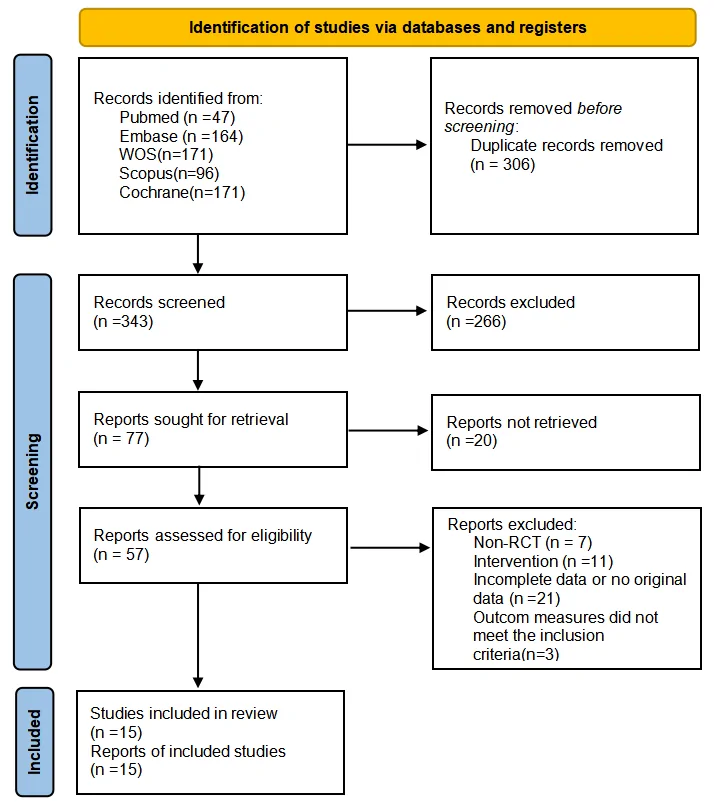
PRISMA (Preferred Reporting Items for Systematic Reviews and Meta-Analyses) flowchart detailing the identification and selection of randomized controlled trials (RCTs) on telehealth interventions for people with Parkinson disease. WOS: Web of Science.

Figure 1 shows the PRISMA flowchart detailing the study selection process for this systematic review and meta-analysis. The initial electronic database search yielded 649 records. After removing duplicates, 343 articles remained. Following title and abstract screening, 266 articles were excluded, leaving 77 articles eligible for full-text review. Among these, 20 articles (conference abstracts, reviews, or research protocols) were unavailable in full, leaving 57 articles for detailed evaluation. Ultimately, 15 articles met the inclusion criteria and were included in the final analysis.

### Study Characteristics

This systematic review included 15 RCTs [20,28-31,34-43] with a total of 765 participants. The studies were conducted across various countries: 2 in Spain, 6 in the United States, 1 in Japan (Tokyo), 3 in Italy, 1 in Sweden, 1 in Brazil, and 1 in Australia. These studies were published between 2016 and 2024. All participants were diagnosed with PD and aged 18 years or older. Sample sizes ranged from 9 to 49 participants. All trials used telehealth technologies, including telehealth systems, telephones, applications, and tablets. Follow-up durations ranged from 1 to 12 months post intervention. Detailed study characteristics are provided in Table 1.

**Table 1:** Characteristics of randomized controlled trials evaluating telehealth interventions for people with Parkinson disease (N=15 studies, 2016-2024).

Author, year	Country	Sample size, T/C^a^	Age (years), T/C^b^, mean (SD)	Participant	Diagnostic criteria	Telehealth technology	Follow-up time	Outcomes (with measure scales)
Cubo et al, 2017 [34]	Spain	17/18	66.44 (7.09)/ 66.05 (9.76)	PD^c^	Medical records	Telehealth system (Kinesia system included a tablet software app, a wireless finger-worn motion sensor unit, and automated web-based symptom reporting.)	12 months	PD severity (UPDRS^d^, parts I-IV), the severity of nonmotor symptoms (Non-Motor Quest), QOL^e^ (EQ-5D), depression and anxiety (HADS^f^), caregiver burden (Zarit Burden)
Del Pino et al, 2023 [35]	Spain	10/10	64.5 (7.9)/ 69.1 (3.5)	PD	UK Brain Bank	Telemedicine system (vCare system: a virtual training platform created based on an intelligent ICT^g^ environment for rehabilitation of neurological and cardiac diseases related to aging)	4 months	QOL (Euroqol 5D), cognitive general status (MoCA^h^), PD severity (UPDRS, parts I-IV), functional disability (H&Y^i^), England ADL^j^
Dobkin et al, 2020 [36]	United States	37/35	65.62 (9.76)/ 64.80 (9.62)	PD and caregivers	National Institute of Neurological Disorders and Stroke research criteria	Telephone (using the telephone for CBT^k^)	End of treatment/6 months	Depression (HAM-D^l^, BDI^m^), anxiety (HAM-A^n^), QOL (SF-36^o^)
Dobkin et al, 2021 [30]	United States	45/45	67.27 (7.79)/66.42 (9.51)	PD	Medical records	Telephone (using the telephone for CBT)	End of treatment/6 months	Depression (HAM-D, BDI), anxiety (HAM-A), QOL (SF-36)
Duffley et al, 2021 [37]	United States	23/19	65.0 (10.9)/64.1 (10.0)	PD and caregivers	Medical records	Telephone (using the telephone for health guidance and follow-up)	6 months	Motor symptoms (UPDRS, part III), QOL (PDQ-39^p^), caregiver burden (MCSI^q^),
Eldemir et al, 2023 [38]	Turkey	15/15	57.87 (9.79)/61.40 (7.29)	PD	UK Brain Bank	Telephone (carrying out rehabilitation training courses through telephone videoconferences)	1.5 months	ADL (UPDRS, part II), motor symptoms (UPDRS, part III), QOL (PDQ-8)
Ellis et al, 2018 [39]	United States	23/21	64.8 (8.5)/63.3 (10.6)	PD	UK Brain Bank	App (Wellpepper app: this health app provides a detailed exercise plan, including what, how, when, and where to perform exercises. Push notifications motivate users to complete their exercise and walking programs. A physical therapist remotely adjusts the regimen based on user progress. Visual progress tracking helps users monitor their performance throughout the program.)	12 months	QOL (PDQ-39), walking capacity (6-MWT^r^)
Gandolfi et al, 2017 [40]	Italy	36/34	67.45 (7.18)/69.84 (9.41)	PD	UK Brain Bank	App (TeleWii-Lab: contains the Nintendo Wii console for motion control input, the Wii Fit game system, and the balance board. A laptop computer connected to a high-definition webcam was used to establish real-time remote video communication between the rehabilitation unit and the patient’s residence via Skype software.)	End of treatment/1 month	QOL (PDQ-8, walking capacity (10-MWT^s^), balance (BBS^t^, ABC^u^)
GoffrEdo et al, 2023 [41]	Italy	49/48	67.8 (6.6)/68.2 (5.8)	PD	UK Brain Bank	Tablet (carry out motor and cognitive rehabilitation training through the VRRS^v^ tablet system based on nonimmersive virtual reality.)	1.5-2.5 months	Walking capacity (6-MWT, TUG^w^), motor symptoms (UPDRS, part III), balance (mini-Balance Evaluation)
Heldman et al, 2017 [42]	United States	9/9	65.2 (10.1)/68.6 (10.2)	PD	Medical records	Telephone (conducting videoconferences or providing telephone guidance for implementing interventions via telephone)	7 months	PD severity (UPDRS, part I-IV), QOL (PDQ-39), the Patient Assessment of Chronic Illness Care
Kraepelien et al, 2020 [29]	Sweden	38/39	65.9 (8.5)/66.1 (9.8)	PD	Medical records	Telephone (using the telephone for CBT)	1.25 months/2.5 months	Depression and anxiety (HADS), QOL (PDQ-8, BBQ^x^)
Maggio et al, 2024 [31]	Italy	T1: 12; T2: 12/10	T1: 59.7 (9.7); T2: 63.8 (8.3)/66.8 (6.5)	PD	Medical records	App (nonimmersive VR^y^ app: NeuroNation Brain Training by Synaptikon GMBH, Berlin, offering science-based mental training to enhance various cognitive abilities with personalized data reports, and Train Your Brain by Grove FX, focusing on specific skills like concentration, spatial thinking, and reasoning. Additionally, there’s a social cognitive app known as Sims Mobile.)	1.5 months/3 months	Cognitive general status (MMSE^z^, MoCa), depression and anxiety (HAM-D)
Pastana Ramos et al, 2023 [28]	Brazil	8/11	60.7 (17.04)/58.6 (8.15)	PD	UK Brain Bank	Tablet\telephone (individualized telerehabilitation sessions were conducted using a tablet or mobile phone through videoconferencing and verbal guidance.)	1 month/2 months	Walking capacity (TUG, 5STS^aa^), balance (ABC), QOL (PDQ-8), motor symptoms (MDS-UPDRS^ab^, part III)
Theodoros et al, 2016 [43]	Australia	15/16	71.62 (7.77)/72.86 (9.99)	PD	Medical records	Telemedicine system (eHAB: a mobile multimedia telerehabilitation system that offers real-time videoconferencing and transmits treatment data to the user’s computer as images and texts. It also records high-definition live video and audio.)	1 month	QOL (PDQ-39)
Wilkinson et a, 2016 [20]	United States	Arm 1: 26/24; Arm 2: 18/18	Arm 1: 76.1 (8.4)/76.1 (7.9); Arm 2: 67.2 (9.8)/70.9 (8.4)	PD	UK Brain Bank	Telemedicine system (a global health care telemedicine specialist cart and Cisco webcam provide real-time high-definition audio-visual connectivity between patients and health care providers.)	6 months/12 months	PD severity (Arm 1: UPDRS, part I-IV; Arm 2: H&Y), QOL (PDQ-8), depression and anxiety (GDS^ac^)

^a^T/C: sample size in treatment group/control group.

^b^T/C: mean (SD) values in treatment group/control group.

^c^PD: Parkinson disease.

^d^UPDRS: Unified Parkinson’s Disease Rating Scale.

^e^QOL: quality of life.

^f^HADS: Hospital Anxiety and Depression Scale.

^g^ICT: digital information and communication technologies.

^h^MoCA: Montreal Cognitive Assessment.

^i^H&Y: Hoehn and Yahr Scale.

^j^ADL: activities of daily living.

^k^CBT: cognitive behavioral therapy.

^l^HAM-D: Hamilton Depression Rating Scale.

^m^BDI: Beck Depression Inventory.

^n^HAM-A: Hamilton Anxiety Rating Scale.

^o^SF-36: Medical Outcomes Study 36-Item Short Form Health Survey.

^p^PDQ: Parkinson Disease Questionnaire.

^q^MCSI: Michigan Consumer Sentiment Index.

^r^6-MWT: 6-minute walk test.

^s^10-MWT: 10-minute walk test.

^t^BBS: Berg Balance Scale.

^u^ABC: Activities-specific Balance Confidence scale.

^v^VRRS: Virtual Router Redundancy Service.

^w^TUG: Timed Up and Go Test.

^x^BBQ: Brunnsviken Brief Quality of Life Scale.

^y^VR: virtual reality.

^z^MMSE: Mini-Mental State Examination.

^aa^5STS: 5 Times Sit-to-Stand Test.

^ab^MDS-UPDRS: Movement Disorders Society-Unified Parkinson’s Disease Rating Scale.

^ac^GDS: Geriatric Depression Scale.

### Risk of Bias

**Figure 2:**
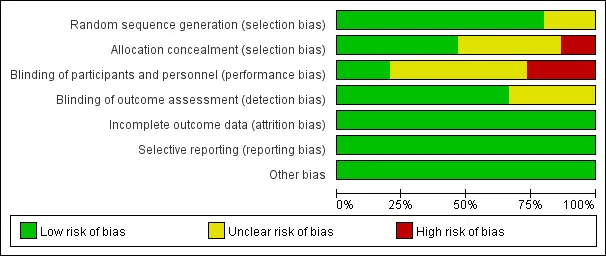
Risk of bias assessment (using the Cochrane Risk of Bias tool) for the included randomized controlled trials evaluating telehealth interventions for Parkinson disease.

The methodological quality and potential biases of the included studies were assessed using the Cochrane Risk of Bias tool [33]. Figure 2 presents detailed findings. All 15 studies were confirmed as randomized, and 12 studies [20,28,30,31,34,36-41,43] clearly described methods of random sequence generation. Only 7 studies [28-30,36,38,39,41] detailed allocation concealment methods, categorizing them as having a low risk of selection bias. Regarding the blinding of participants and intervention providers, only 3 studies [36,39,41] reported adequate blinding procedures and thus had a low risk of performance bias. In contrast, 4 studies [28,37,40,43] without blinding were classified as having a high risk of performance bias. The remaining 8 studies [20,29-31,34,35,38,42] lacked sufficient information and were classified as unclear. Blinding of outcome assessors was reported in 10 studies [28,30,36-43], indicating a low risk of detection bias, while 5 studies [20,29,31,34,35] did not report this information. All studies provided complete outcome data, indicating low attrition bias. Assessment for other potential biases generally indicated low risk. Funnel plot analysis (Multimedia Appendix 4) revealed no significant publication bias among included studies.

### Meta-Analysis

#### Quality of Life

A total of 11 studies [20,28-30,36-40,42,43] evaluated QOL following telehealth interventions, using 5 different measurement tools. Overall, 9 studies used Parkinson Disease Questionnaire (PDQ) scales (PDQ-39 [37,39,42,43] and PDQ-8 [20,28,29,38,40]), with higher scores indicating worse QOL. Additionally, 2 studies used the Medical Outcomes Study 36-Item Short Form Health Survey (SF-36) [30,36], and 1 study incorporated both PDQ-8 and Brunnsviken Brief Quality of Life Scale (BBQ) [29], with higher scores reflecting better QOL. Due to measurement variability, a standardized mean difference (SMD)–based meta-analysis was conducted following Cochrane handbook guidelines.

A 3-level random-effects meta-analysis of 8 studies (13 effect sizes) assessing QOL using PDQ scales revealed a marginal trend toward improvement after telehealth interventions (SMD –0.42, 95% CI –0.88 to 0.03; *P*=.07). Heterogeneity was present (τ^2^=0.258; *I*^2^=56.2%; Q_12_=27.37; *P*=.007), with between-study variance accounting for 65.5% of total variability. Prediction intervals were not calculated due to the limited number of studies (*k*<10; Table 2; Figures S1-S3 in Multimedia Appendix 4). Subgroup analysis demonstrated significant moderation by intervention type (test of moderators=9.84; *P*=.002). Telephone-based interventions significantly improved QOL (SMD –0.83, 95% CI –1.22 to –0.44; *P*<.001), whereas digital interventions had minimal effect (SMD –0.05, 95% CI –0.58 to 0.49; *P*=.86). The between-group difference was 0.78 (95% CI 0.29-1.27; *P*=.002) points. Follow-up duration did not significantly moderate outcomes (categorical: *P*=.58; continuous: β=.01/month; *P*=.83; Table 3; Figures S4-S7 in Multimedia Appendix 4). Sensitivity analysis identified substantial influence from Ramos et al [28], whose exclusion reduced the effect magnitude by 52.2%. Multilevel Egger test suggested possible small-study effects (*P*=.02; Figures S8-S9 in Multimedia Appendix 4).

**Table 2:** Results of 3-level random-effects meta-analyses for primary outcomes in patients with Parkinson disease receiving telehealth interventions.

Outcome	*k* ^a^	*#ES* ^b^	Mg^c^	95% CI	*P* value	σ^2^_level2_^d^	σ^2^_level3_^e^	Variance level 1 (%)	Variance level 2 (%)	Variance level 3 (%)
QOL^f,g^	8	13	–0.42	–0.88 to 0.034	.07	0.000	0.258	34.5	0	65.5
QOL^h^	3	5	0.39	0.06 to 0.72	.03	0.019	0.000	72.3	27.7	0
Anxiety	3	5	–0.64	–0.92 to –0.35	.003	0.000	0.000	100	0	0
Cognitive function	2	9	1.12	0.03 to 2.20	.045	0.192	0.275	33.9	27.2	38.7
Depression	5	17	–0.64	–0.93 to –0.34	＜.001	0.016	0.062	55.4	8.9	35.6
Motor symptoms	7	11	–0.46	–0.69 to –0.24	＜.001	0.000	0.000	100	0	0

^a^*k*=number of studies.

^b^*#ES*=number of effect sizes.

^c^Mg=mean effect size (g).

^d^σ^2^_level2_=variance between effect sizes extracted from the same study.

^e^σ^2^_level3_=variance between studies.

^f^QOL: quality of life.

^g^The 3-level meta-analysis of 8 studies (13 effect sizes) assessing QOL with Parkinson Disease Questionnaire.

^h^The 3-level meta-analysis of 3 studies (5 effect sizes) assessing QOL with Medical Outcomes Study 36-Item Short Form Health Survey and Brunnsviken Brief Quality of Life Scale.

**Table 3:** Moderator analyses examining the effects of intervention type and follow-up duration on primary outcomes in the meta-analysis.

		*k* ^a^	*#ES* ^b^	B_0_/_g_^c^	t_0_^d^	B_1_^e^	t_1_^f^	*F* test (*df*)^g^
**QOL^h,i^**								
	Intervention type	8	13	–0.83	–4.179^j^	0.78	3.137^j^	9.842^j^ (1, 11)
	Follow duration^k^	8	13	–0.53	–1.852	0.26	0.550	0.302 (1, 11)
	Follow duration^l^	8	13	–0.46	–0.167	0.01	0.219	0.048 (1, 11)
**QOL^m^**								
	Follow duration^k^	3	5	0.73	3.033^j^	–0.42	–0.157	2.470 (1, 3)
	Follow duration^l^	3	5	0.69	3.369^n^	–0.06	–1.713	2.937 (1, 3)
**Anxiety**								
	Follow duration^k^	3	5	–0.51	–2.206^o^	–0.16	–0.605	0.366 (1, 3)
	Follow duration^l^	3	5	–0.66	–3.179^j^	0.004	0.124	0.015 (1, 3)
**Cognitive function**								
	Follow duration^k^	2	9	1.39	2.876^j^	–0.31	–0.654	0.427 (1, 7)
	Follow duration^l^	2	9	2.07	3.125^j^	–0.30	–0.186	1.407 (1, 7)
**Depression**								
	Intervention type	5	17	–0.84	–9.933^n^	0.53	3.416^n^	11.669^n^ (1, 15)
	Follow duration 1^k^	5	17	–0.54	–1.979^o^	–0.13	–0.419	0.175 (1, 15)
	Follow duration 2^l^	5	17	^—^0.87	–4.798^n^	0.04	1.860	0.346 (1, 15)
**Motor symptoms**								
	Intervention type	7	11	—0.54	—3.542^n^	0.13	0.660	0.436 (1, 9)
	Follow duration^k^	7	11	–0.58	–3.624^n^	0.20	0.949	0.901 (1, 9)
	Follow duration^l^	7	11	–0.55	–3.037^j^	0.015	0.595	0.354 (1, 9)

^a^k=number of independent studies.

^b^*#ES=*number of effect sizes.

^c^B_0_/mean g=intercept/mean effect size (g).

^d^t_0_=*t* value for mean g.

^e^B_1_=estimated regression coefficient.

^f^t_1_=*t* value for regression coefficient.

^g^Omnibus *F* test.

^h^QOL: quality of life.

^i^The 3-level meta-analysis of 8 studies (13 effect sizes) assessing QOL with Parkinson Disease Questionnaire.

^j^*P*<.01.

^k^Results of analysis based on categorical variables.

^l^Results of analysis based on continuous variables.

^m^The 3-level meta-analysis of 3 studies (5 effect sizes) assessing QOL with the Medical Outcomes Study 36-Item Short Form Health Survey and Brunnsviken Brief Quality of Life Scale.

^n^*P*<.001.

^o^*P*<.05.

For 3 studies assessing QOL using SF-36 and BBQ (5 effect sizes), the 3-level random-effects meta-analysis indicated significant improvement after telehealth interventions (SMD 0.39, 95% CI 0.06-0.72; *P*=.03). Minimal heterogeneity was observed (τ^2^=0.020; *I*^2^=25.9%; Q_4_=5.40; *P*=.25), with within-study variance contributing 27.7% of total variability. Prediction intervals were not calculated (*k*<10; Table 2; Figures S11-S12 in Multimedia Appendix 4). Subgroup analyses indicated greater effects in short-term follow-ups (<3 months: SMD 0.73, 95% CI 0.26-1.19) compared with longer-term follow-ups (≥3 months: SMD 0.31, 95% CI –0.40 to 0.43; β=–.42; *P*=.12). A marginal negative correlation between effect magnitude and follow-up duration was observed (β=–.06/month; *P*=.09; Table 3; Figures S13-S15 in Multimedia Appendix 4). Sensitivity analysis confirmed robustness; no single-study exclusion altered significance substantially (maximum change: +33.2% when excluding Dobkin et al [36]). The Egger test indicated no significant small-study effects (*P*=.28; Figures S16-S17 in Multimedia Appendix 4).

#### Depression

A total of 5 studies [20,29-31,36] evaluated depression levels in patients with PD following telehealth interventions, using 4 distinct assessment tools: Hamilton Depression Rating Scale (HAM-D) [30,31,36], Beck Depression Inventory (BDI) [30,36], Hospital Anxiety and Depression Scale-Depression (HADS-D) [29], and Geriatric Depression Scale [20]. Higher scores on these scales indicate increased depression severity. Two studies used both the HAM-D and BDI to assess depression. A 3-level random-effects meta-analysis (5 studies, 17 dependent effect sizes) with t‑distribution–based inference revealed a significant reduction in depression symptoms after telehealth interventions (SMD –0.64, 95% CI –0.93 to –0.34; *P*<.001). Moderate heterogeneity was observed (*I*^2^=45.9%; Q_16_=29.60; *P*=.02). Variance component analysis indicated that between-study differences accounted for 35.6% of total variability (τ^2^=0.062), within-study variability explained 8.9% (τ^2^=0.016), and sampling error contributed 55.4% (Table 2; Figures S17-S19 in Multimedia Appendix 4). Prediction intervals were not calculated because the number of studies was below the recommended threshold (*k*<10). Subgroup analysis showed significantly greater improvements with traditional telehealth (SMD –0.84, 95% CI –1.00 to –0.67; *P*<.001) compared with digital interventions (SMD –0.31, 95% CI –0.54 to –0.08; *P*=.008; β=.53; *P*<.001). Follow-up duration was not significant in categorical analysis (β=–.13; *P*=.68), but continuous analysis showed a marginal positive association (β=.04/month; *P*=.06; Table 3; Figures S20-S23 in Multimedia Appendix 4). Sensitivity analyses confirmed robustness; effects remained significant after excluding each study (SMD range –0.55 to –0.79). The Egger test indicated no significant small-study effects (*P*=.82; Figures S24-S25 in Multimedia Appendix 4).

#### Anxiety

A total of 3 studies [29,30,36] assessed anxiety levels in patients with PD using 2 measurement scales: Hamilton Anxiety Rating Scale (HAM-A) [30,36] and Hospital Anxiety and Depression Scale-Anxiety (HADS-A) [29]. Higher scores represent greater anxiety severity. A 3-level random-effects meta-analysis (3 studies, 5 dependent effect sizes) with t-distribution–based inference indicated significant anxiety reduction following telehealth interventions (SMD –0.64, 95% CI –0.92 to –0.35; *P*=.003). Negligible heterogeneity was observed (*I*^2^=0%; Q_4_=0.90; *P*=.92), with variance component analysis indicating that sampling error explained all variability (τ^2^=0 for both inter- and intrastudy variance; Table 2; Figures S26-S28 in Multimedia Appendix 4). Prediction intervals were not calculated due to the limited number of studies (*k*<10). All studies used telephone-based interventions, thus subgroup analyses examined follow-up durations. Follow-up duration was not significantly associated with outcomes in categorical (β=–.16, 95% CI –0.66 to 0.35; *P*=.55) or continuous analyses (β=.004/month, 95% CI –0.06 to 0.07; *P*=.90; Table 3; Figures S29-S31 in Multimedia Appendix 4). Sensitivity analyses confirmed robustness, with effects remaining significant upon exclusion of individual studies (SMD range –0.67 to –0.60), and maximum deviation of –5.4% when excluding Dobkin et al [30]. Funnel plots were symmetrical, and the Egger test revealed no significant small-study effects (*P*=.68; Figures S32-S33 in Multimedia Appendix 4).

#### Motor Symptoms

A total of 7 studies [20,28,34,35,38,41,42] used the MDS-UPDRS-III to evaluate motor symptoms in patients with PD post telehealth interventions, with higher scores indicating greater severity. A 3-level random-effects meta-analysis (7 studies, 11 dependent effect sizes) with t‑distribution–based inference demonstrated significant improvements in motor symptoms after telehealth interventions (SMD –0.46, 95% CI –0.69 to –0.24; *P*=.001). Heterogeneity among studies was negligible (*I*^2^=0%; Q_10_=7.85; *P*=.64). Variance component analysis showed that sampling error accounted for all variability, with negligible between-study (τ^2^=0) and within-study variance (τ^2^=0; Table 2; Figures S34-S36 in Multimedia Appendix 4). Prediction intervals were not calculated because the number of studies was below the recommended threshold (*k*<10). Subgroup analysis by intervention type revealed no significant difference between digital and other telehealth interventions (β=.13, 95% CI –0.26 to 0.53; *P*=.51). Follow-up duration did not significantly moderate outcomes in categorical (β=.20, 95% CI –0.21 to 0.60; *P*=.34) or continuous analyses (β=.02/month, 95% CI –0.03 to 0.06; *P*=.55; Table 3; Figures S37-S39 in Multimedia Appendix 4). Sensitivity analyses confirmed robustness, with significant effects maintained after each study’s exclusion (SMD range –0.52 to –0.42), and the largest change being 11.5% when excluding Wilkinson et al [20]. Funnel plots showed symmetry, and the Egger test indicated no significant small-study effects (*P*=.88; Figures S40-S41 in Multimedia Appendix 4).

#### Activities of Daily Living

A total of 4 studies [34,35,38,42] evaluated the impact of telehealth interventions on daily activities in patients with PD using the MDS-UPDRS-II scale. Higher scores represent greater impairment. A random-effects meta-analysis with HKSJ correction demonstrated that telehealth interventions significantly reduced impairment in daily activities compared with controls (SMD –0.79, 95% HKSJ-adjusted CI –1.04 to –0.54; *P*=.002). Heterogeneity was negligible (τ^2^=0.000; *I*^2^=0.0%; Q_3_=0.43; *P*=.93). Prediction intervals were not calculated because the number of studies was below the recommended threshold (*k*<10). Sensitivity analysis (leave-one-out) confirmed the robustness of the findings. The largest change in effect size occurred after excluding Del Pino et al [35] (–7.9% change; SMD range across exclusions –0.83 to –0.73). Egger test indicated no significant small-study effects (intercept=–1.35; *P*=.31; Figure S42 in Multimedia Appendix 4).

#### Cognition

A total of 2 studies [31,35] examined cognitive outcomes in patients with PD after telehealth interventions using the Montreal Cognitive Assessment (MoCA) and Mini-Mental State Examination (MMSE), where higher scores indicate better cognitive function. One study used both the MoCA and MMSE scales to comprehensively assess cognition. A 3-level random-effects meta-analysis (2 studies, 9 dependent effect sizes) with t‑distribution–based inference indicated significant cognitive improvement following telehealth interventions (SMD 1.12, 95% CI 0.03-2.20; *P*=.045). Moderate heterogeneity was detected (*I*^2^=52.3%; Q_8_=16.77; *P*=.03). Variance component analysis showed that between-study differences accounted for 38.9% (τ^2^=0.275) of total variability, within-study differences accounted for 27.2% (τ^2^=0.192), and sampling error explained 33.9% (Table 2; Figures S43-S45 in Multimedia Appendix 4). Prediction intervals were not calculated due to the limited number of studies (*k*<10). A follow-up duration did not significantly moderate outcomes in categorical (β=–.31, 95% CI –1.25 to 0.62; *P*=.51) or continuous analysis (β=–.30/month, 95% CI –0.79 to 0.20; *P*=.24; Table 3; Figures S46-S48 in Multimedia Appendix 4). The Egger test indicated significant small-study effects (intercept=15.17; *P*<.001). Sensitivity analysis was not feasible due to the limited number of studies (Figure S49 in Multimedia Appendix 4).

## Discussion

### Overview

This systematic review evaluated the effects of telehealth interventions on the QOL and associated health outcomes in patients with PD. Findings demonstrated that telehealth interventions significantly enhanced various dimensions of patient well-being, including QOL, depressive symptoms, anxiety levels, motor function, ADL, and cognitive abilities. These results differ from earlier reviews and meta-analyses [8,26,27]. The discrepancies might be due to accelerated advancements in telehealth and remote neurology after the epidemic, alongside improvements in telehealth service quality and increased research volume [22,44]. Although the effectiveness of telehealth interventions appears promising, additional studies are needed to establish more conclusive evidence.

### Effectiveness of Telehealth Interventions on QOL in Patients With PD

Our meta-analysis identified complex patterns regarding telehealth’s effect on QOL in patients with PD. Interventions assessed by the SF-36/BBQ demonstrated a significant improvement, whereas those assessed using PDQ scales indicated only marginal benefit. This difference likely stems from the fundamental distinctions between scales: PDQ scales specifically measure PD-related deficits, whereas SF-36/BBQ assess general well-being [45,46]. Notably, telephone-based interventions substantially improved PDQ-based QOL, while digital interventions showed negligible effects. This distinction explains inconsistencies in prior meta-analyses [1,26,27] that did not account for intervention modality. Significant heterogeneity in PDQ analyses was primarily attributed to between-study differences, suggesting methodological variations. Sensitivity analysis highlighted substantial influence from Ramos et al [28], and potential small-study effects were noted. These factors necessitate cautious interpretation of PDQ outcomes. Conversely, SF-36/BBQ analyses exhibited minimal heterogeneity and robust sensitivity. Differences in results may also reflect variations in intervention type, therapeutic intensity, assessment timing, methodological quality, and sample sizes [27]. Follow-up duration did not moderate effects, contradicting assumptions that longer interventions yield superior outcomes. Instead, the intervention modality emerged as the critical moderator, highlighting the importance of direct human interaction in managing PD-specific QOL concerns. This aligns with telehealth’s capacity for dynamic therapeutic engagement [47,48], particularly beneficial for isolated patients [10], although our findings indicate that these advantages depend on modality.

Telehealth provides more dynamic, immersive methods for treatment, education, and counseling compared to traditional medical approaches, enhancing patient engagement and interaction [47,48]. Such enhancements assist patients with PD and their families in comprehensively understanding and managing disease-related challenges, thus promoting independence, motivation for self-care, and improved life quality [49]. Patients with PD require consistent engagement with health care teams for effective management of disease progression and treatment complexity [8]. A primary advantage of telehealth lies in serving patients in isolated or underserved areas, addressing health care provider shortages, and offering timely, high-quality care to improve patient outcomes [10]. Additionally, economic burdens and logistical difficulties substantially reduce patients with PD’s QOL [50,51]. By reducing health care–related costs and travel demands, telehealth can expand home-based medical services, further enhancing life quality for patients with PD [10,22].

### Effectiveness of Telehealth Interventions on Depression in Patients With PD

Our meta-analysis demonstrated a significant antidepressant effect of telehealth interventions. Traditional approaches (telephone or cognitive behavioral therapy [CBT]–based) showed nearly 3 times the efficacy compared to digital interventions. This advantage aligns with neurobiological evidence linking depression in PD to dysfunction in serotonergic pathways and frontostriatal circuits [52], suggesting human-mediated therapies more effectively modulate emotional processing compared to automated digital tools. This finding notably diverges from earlier studies, which primarily emphasized motor symptoms and physical rehabilitation, often neglecting depressive symptoms [10,27]. The distinct focus of 3 specific studies [29,30,36] included in this meta-analysis may explain this difference. These studies emphasized cognitive and behavioral aspects of patient care, integrating telehealth with CBT, a method recognized for effectively reducing depression levels [53,54]. Moderate heterogeneity mainly resulted from between-study methodological differences, likely reflecting variations in measurement tools and intervention protocols. Contrary to expectations, categorical follow-up duration showed no significant moderating effect, although continuous analysis revealed a marginal positive association. This result suggests sustained engagement—particularly through telephone-based CBT [53,54]—might progressively reinforce neuroplastic changes in emotion-regulation networks. These findings reconcile previous contradictions in the literature [1,26,27]. Whereas earlier reviews primarily targeted motor symptoms, our analysis confirms telehealth’s antidepressant benefits when including behavioral interventions tailored to PD-related psychopathology.

Our findings align with those of Dou et al [55], indicating that telehealth interventions (tele-CBT and telerehabilitation training) significantly improve depressive symptoms in patients with PD. Feasibility has been verified internationally; for example, a cross-sectional study in Brazil showed effective telehealth implementation in resource-limited settings with high patient satisfaction [56,57]. Regarding specific methods, tele-motor training significantly enhanced patients’ motor function and indirectly alleviated depressive symptoms [55]. In contrast, tele-CBT directly targeted depressive and anxiety symptoms, showing greater effectiveness compared to other teleinterventions [31]. Teleconsultation had relatively limited efficacy in alleviating depressive symptoms but significantly improved access to medical resources [58]. From a neurobiological standpoint, PD and depression share common pathological mechanisms, including gut microbiota dysregulation, neuroinflammation, and reward-processing dysfunction [52]. Telehealth, especially through behavioral interventions such as CBT, may modulate these pathological processes and consequently alleviate depressive symptoms [59]. However, existing evidence suggests telehealth’s effectiveness may be weaker for chronic, nonepisodic mental disorders (eg, depression in PD) compared to primary depression [60]. To optimize telehealth potential, future research should investigate long-term outcomes, standardization of techniques, and cybersecurity considerations [61,62]. In summary, telehealth effectively reduces depressive symptoms in patients with PD, especially via tele-CBT, which overcomes geographical barriers and improves treatment accessibility. Nevertheless, individualized plans and sustained follow-up are necessary to achieve optimal therapeutic outcomes.

### Effectiveness of Telehealth Interventions on Anxiety in Patients With PD

Our meta-analysis demonstrated robust anxiolytic effects of telephone-based telehealth interventions, with remarkable consistency across studies. This homogeneity suggests that telephone-delivered CBT provides a reliably standardized approach for managing PD-related anxiety. Notably, these benefits remained stable irrespective of follow-up duration, indicating sustained therapeutic effects without attenuation over 3-9 months. These findings resolve previous contradictions [14,26,27] by demonstrating that structured tele-CBT can effectively address PD-specific anxiety mechanisms, including fear-avoidance cycles and “off”-period distress resistant to conventional treatments. The negligible heterogeneity, with variance entirely attributable to sampling error, likely reflects 3 factors. First, interventions used standardized CBT protocols targeting PD-specific anxiety mechanisms such as hypervigilance toward motor fluctuations. Second, the uniform application of validated and sensitive scales (HAM-A/HADS-A) ensured measurement precision. Third, telephone delivery strengthened therapeutic alliances through real-time emotional interaction absent in purely digital interfaces. The integration of standardized protocols, precise assessments, and person-centered delivery resulted in methodological consistency across studies.

Although considerable evidence supports telehealth for anxiety in patients with PD, its exact mechanism and broader applicability require further investigation. Previous studies [63,64] showed comparable efficacy of telehealth and face-to-face interventions in reducing anxiety, depression, and stress scores, alongside improved heart rate variability. Anxiety reductions persisted long-term after telehealth interventions, confirming their noninferiority to in-person care. This effectiveness largely stems from multimodal interventions; for example, remote CBT overcomes movement-related barriers and, combined with exercise and biomarker monitoring, allows personalized care beneficial to underserved populations [55,64,65]. However, some research highlights intervention heterogeneity. A small study [66] indicated that telephone CBT effectively alleviated depression but not anxiety symptoms in patients with PD, suggesting anxiety may require more tailored strategies. Our analysis, in contrast, supports the long-term feasibility, effectiveness, and durability of telephone CBT effects. Earlier discrepancies might stem from small sample sizes or limitations of measurement tools. Although the revised Parkinson Anxiety Scale improved cultural adaptability, general scales (eg, HADS) may underestimate actual effectiveness due to limited sensitivity [29,67]. Future research should expand sample sizes, develop PD-specific anxiety interventions, and integrate multidimensional biomarker monitoring to improve telehealth precision and applicability.

### Effectiveness of Telehealth Interventions on Motor Symptoms of Patients With PD

Telehealth interventions significantly improved motor symptoms in patients with PD. This refined estimate may reflect advancements in methodological rigor involving multilevel analyses that account for independent effect sizes, an approach not consistently used in previous meta-analyses [1,26]. Due to the standardized use of MDS-UPDRS-III assessments and similar intensities of interventions, we observed remarkably low heterogeneity among studies. Notably, digital and traditional telehealth approaches demonstrated comparable effectiveness, indicating that essential motor rehabilitation components, such as amplitude training and balance exercises, effectively translated across different treatment platforms. The temporal stability of benefits further supported telehealth as a sustainable management option, with sensitivity analyses confirming robustness to study exclusion.

A primary therapeutic objective in PD involves improving motor symptoms, wherein treatment adjustments frequently depend on accurate motor assessments [68]. The telehealth framework enables improved and timely interactions between patients and health care providers compared to traditional face-to-face consultations, allowing for more individualized rehabilitation strategies tailored specifically to patients with movement disorders [69]. Multiple studies have confirmed the significant impact of telehealth interventions in alleviating motor symptoms in patients with PD. For instance, structured telerehabilitation programs, such as the Lee Silverman Voice Treatment BIG rehabilitation method, have effectively enhanced motor function, alleviated nonmotor symptoms, and improved the QOL for patients with PD [70]. Compared to teleconsultations alone, tele-motor interventions demonstrate superior efficacy in motor function improvement [55]. From a neuromechanism perspective, cueing techniques activate the motor cortex, thereby enhancing the stability of motor output, which provides scientific justification for using cue-based strategies in telerehabilitation [71]. Moreover, telerehabilitation is particularly suitable for patients with restricted mobility or those residing in medically underserved regions. Real-time video guidance ensures continuous rehabilitation training, effectively overcoming geographical limitations. Its safety and potential effectiveness in improving balance and functional activities have been confirmed by existing research [28,41,72]. Telehealth facilitates comprehensive monitoring of treatment effects through standardized scales (such as MDS-UPDRS) for assessing motor symptoms, combined with evaluation of nonmotor symptoms and QOL questionnaires [73,74]. Additionally, tele-motor interventions based on live-streaming have been proven feasible and safe, demonstrating high patient adherence (eg, twice a week) and thus confirming their practical use for continuous management of motor symptoms in PD [75]. Therefore, telehealth effectively enhances motor functions in patients with PD, offering advantages in personalized program design, activation of neural plasticity, and overcoming limitations in medical resource availability. With ongoing advancements in assessment instruments and technological integration, telehealth is anticipated to further improve long-term intervention outcomes.

### Effectiveness of Telehealth Interventions on ADL in Patients With PD

The results of this study showed that telehealth interventions significantly improved ADL among patients with PD. This result aligns with previous research, reinforcing that remote health care interventions significantly enhance both ADL performance and motor symptoms in individuals with PD. Our analysis suggests that the significant improvements in ADL resulting from telehealth are due to multidimensional intervention strategies addressing the core symptoms of PD.

Relevant studies have shown that structured remote rehabilitation programs, delivered through real-time video instruction, enhance functional mobility and directly improve basic ADL tasks such as walking and dressing [57]. Simultaneously, high-intensity remote exercise interventions reduce motor sluggishness and freezing of gait, indirectly enhancing instrumental ADLs, such as complex daily activities like shopping and meal preparation [76]. The simultaneous improvements observed in ADLs and motor symptoms share clear pathophysiological connections; enhanced motor functions directly alleviate limitations in physical activity, enabling patients to execute daily routines more effectively [77]. Additionally, remote CBT improves executive functions, mitigating motor-related restrictions on complex ADL performance [77,78]. Telehealth frameworks achieve these synergistic effects by integrating 3 primary components: real-time video supervision ensures adherence to exercise regimens; home-based cognitive training modules restructure the prefrontal-limbic circuitry; and wearable sensors provide immediate feedback regarding movement quality [57,79,80]. Therefore, telehealth interventions positively and synergistically influence both motor symptoms and ADL performance in patients with PD. Future research should focus on optimizing intervention strategies and integrating motor and ADL training components comprehensively to further enhance the overall QOL for patients.

### Effectiveness of Telehealth Interventions on Cognition in Patients With PD

Preliminary evidence shows that telehealth interventions may enhance cognitive function in patients with PD; however, these findings should be interpreted cautiously. Although statistically significant, effect sizes exhibited substantial variability, ranging from negligible to considerable clinical improvement. This observed heterogeneity primarily stems from methodological differences among studies, potentially reflecting (1) the use of varied cognitive assessments (MoCA vs MMSE), each with differing sensitivities to PD-specific cognitive deficits, and (2) distinct intervention protocols within the limited scope of available evidence. Additionally, significant publication bias and insufficient data for sensitivity analyses further limit definitive conclusions.

Overall, the efficacy of telehealth interventions for enhancing cognitive functions in patients with PD has been established. These interventions significantly improve cognitive status, particularly executive functions and memory, as well as emotional and behavioral disorders, consequently enhancing the QOL for both patients and caregivers [78,81]. Among specific intervention methods, computer-assisted cognitive training has shown potential benefits for patients with PD accompanied by mild cognitive impairment, with feasibility confirmed for home-based training modalities [82,83]. Moreover, remote virtual reality applications (telehealth virtual reality) have shown promising results for improving cognitive task performance [31]. A recent network meta-analysis further supports the beneficial effects of remote interventions, including remote cognitive training, on cognition and other nonmotor symptoms [55]. The primary advantages of telehealth interventions include high accessibility (especially beneficial for patients with limited mobility or those residing in remote areas) and flexibility, with the patient’s cognitive reserve potentially enhancing treatment effect [84]. However, considerable heterogeneity exists within current evidence, aligning with our findings. This heterogeneity is largely attributed to variations in study design, inconsistencies in cognitive assessment tools, and diverse responses among patient subtypes [55,82,85]. In addition, the efficacy of telehealth interventions differed across cognitive domains. Therefore, these findings should be considered exploratory and interpreted cautiously. In conclusion, telehealth represents a promising cognitive management approach for PD with substantial potential; nevertheless, implementation barriers must be considered, strategies tailored to individual patient needs, and larger standardized trials conducted to further substantiate effectiveness.

### Strengths and Limitations

This systematic review benefits from a rigorous methodological approach, using a meta-analysis grounded in RCTs and strictly adhering to established guidelines for systematic reviews. All analyses were conducted using random-effects models based on conceptual considerations, with HKSJ or t-distribution–based corrections applied to provide more accurate and conservative CIs. This significantly enhances the credibility of the findings. Furthermore, the review assesses the impact of telehealth interventions not only on QOL but also on multiple health-related domains such as depression, anxiety, motor function, ADL, and cognitive function in patients with PD, rather than restricting its focus solely to treatment modalities.

Nonetheless, several limitations of this review should be acknowledged. First, the limited number of RCTs included in the analysis may constrain the generalizability of these conclusions to broader populations. Second, due to the small number of studies per outcome (all *k*<10), prediction intervals were not calculated, which limits the interpretation of how the true effect may vary across different settings. Third, funnel plots and Egger tests were used to assess small-study effects, but these methods have reduced accuracy when fewer than 10 studies are analyzed per outcome, and they do not specifically measure publication bias. Finally, although we applied multilevel modeling to account for dependent effect sizes, residual heterogeneity, and variations in intervention protocols may still influence the results. Therefore, additional RCTs must be incorporated into future research to enhance the robustness and reliability of the findings.

### Implications for Practice

Global disparities in medical resource distribution present substantial challenges to health care service advancement. This issue is particularly pronounced in neurological care, where specialist availability is limited, notably in suburban and rural regions. Consequently, many individuals with PD struggle to receive continuous medical support, resulting in significant declines in their QOL as the disease progresses. This situation places considerable strain not only on patients and their families but also on societal resources. The emergence of telehealth, however, offers a promising solution by providing innovative avenues for managing and treating PD. Telehealth has the potential to bridge existing gaps, enabling patients with PD who previously had limited or no access to receive essential health care services.

### Implications for Further Research

PD exerts substantial impacts on public health, prompting significant attention from the health care community toward preventative, diagnostic, and therapeutic strategies. As an innovative product of rapid technological advancement, telehealth represents a cost-effective, real-time, and secure platform for collecting patient data, significantly facilitating the diagnosis, monitoring, and rehabilitation of PD. Nonetheless, the efficacy of telehealth requires further validation through comprehensive and rigorous RCTs. Future research should not only evaluate functional recovery, cognitive enhancement, and health-related QOL but also examine aspects such as cost-effectiveness, patient satisfaction, and digital health literacy among older adults. Such investigations will facilitate more informed decisions and optimal tailoring of telehealth interventions for patients with PD.

### Conclusion

Telehealth interventions have demonstrated the potential to significantly enhance various aspects of life among patients with PD, including alleviating symptoms of depression and anxiety, improving motor function, facilitating ADL, and enhancing cognitive performance. Despite these encouraging findings, there remains an urgent need for meticulously designed, large-scale RCTs to comprehensively evaluate telehealth’s effectiveness across the full spectrum of PD management.

## Data Availability

All data generated or analyzed during this study are included in this published article and its supplementary information files.

## Appendix

Multimedia Appendix 1 PRISMA 2020 checklist.

Multimedia Appendix 2 Search strategy.

Multimedia Appendix 3 R language code.

Multimedia Appendix 4 Supplementary figures.

## Abbreviations

ADL: activities of daily living
BBQ: Brunnsviken Brief Quality of Life Scale
BDI: Beck Depression Inventory
CBT: cognitive behavioral therapy
HADS-A: Hospital Anxiety and Depression Scale-Anxiety
HADS-D: Hospital Anxiety and Depression Scale-Depression
HAM-A: Hamilton Anxiety Rating Scale
HAM-D: Hamilton Depression Rating Scale
HKSJ: Hartung-Knapp-Sidik-Jonkman
MDS-UPDRS: Movement Disorders Society-Unified Parkinson’s Disease Rating Scale
MMSE: Mini-Mental State Examination
MoCA: Montreal Cognitive Assessment
PD: Parkinson disease
PDQ: Parkinson Disease Questionnaire
PRISMA: Preferred Reporting Items for Systematic Reviews and Meta-Analyses
QOL: quality of life
RCT: randomized controlled trial
SF-36: Medical Outcomes Study 36-Item Short Form Health Survey
SMD: standard mean difference
