# Correction: Effects of Telehealth Interventions for People With Parkinson Disease: Systematic Review and Meta-Analysis of Randomized Controlled Trials

**DOI:** 10.2196/108495

**Published:** 2026-09-01

**Authors:** Minyue Sun, Fuyou Tang, Luo min, Shiyu Wen, Shuang Wang, Huiping Jiang

**Affiliations:** 1 Department of Neurology Mianyang Central Hospital, School of Medicine University of Electronic Science and Technology of China Mianyang China; 2 Mianyang Key Laboratory of Anesthesia and Neuroregulation, Department of Anesthesiology Mianyang Central Hospital, School of Medicine University of Electronic Science and Technology of China Mianyang, Sichuan China; 3 Department of Neurology Mianyang Central Hospital, School of Medicine University of Electronic Science and Technology of China Mianyang, Sichuan China

In “Effects of Telehealth Interventions for People With Parkinson Disease: Systematic Review and Meta-Analysis of Randomized Controlled Trials” [1], the designation for co–first authors was inadvertently omitted.

The author list was revised by adding an asterisk next to authors MS and FT and a footnote indicating their equal contribution, as follows:

Minyue Sun^1*^, MSN; Fuyou Tang^2*^, MSN; Luo min^1^, BS; Shiyu Wen^1^, BS; Shuang Wang^3^, BS; Huiping Jiang^3^, BS

*these authors contributed equally

All other aspects of the article remain unchanged.

The correction will appear in the online version of the paper on the JMIR Publications website, together with the publication of this correction notice. Because this was made after submission to PubMed, PubMed Central, and other full-text repositories, the corrected article has also been resubmitted to those repositories.

## References

[1] Sun Minyue, Tang Fuyou, Min Luo, Wen Shiyu, Wang Shuang, Jiang Huiping (2026). Effects of telehealth interventions for people with Parkinson disease: systematic review and meta-analysis of randomized controlled trials. JMIR Mhealth Uhealth.

