# Supplementary material for: Effects of Telehealth Interventions for People With Parkinson Disease: Systematic Review and Meta-Analysis of Randomized Controlled Trials
Source: JMIR Mhealth Uhealth. 2026 Jan 28;14:e70994. doi: 10.2196/70994 (PMC12895161; doi:10.2196/70994)
Supplement: Multimedia Appendix 2 [file mhealth_v14i1e70994_app2.doc]

**Search strategy**

**Pubmed**

("Parkinson Disease"[MeSH Terms] OR ("idiopathic parkinson s disease"[Title/Abstract] OR "lewy body parkinson s disease"[Title/Abstract] OR "parkinson s disease idiopathic"[Title/Abstract] OR "parkinson s disease lewy body"[Title/Abstract] OR "paralysis agitans"[Title/Abstract] OR "parkinson s disease"[Title/Abstract] OR "idiopathic parkinson disease"[Title/Abstract] OR "lewy body parkinson disease"[Title/Abstract] OR "primary parkinsonism"[Title/Abstract] OR "parkinsonism primary"[Title/Abstract] OR "parkinson disease idiopathic"[Title/Abstract] OR "parkinson s disease idiopathic"[Title/Abstract] OR "idiopathic parkinsonism"[Title/Abstract] OR (("lewy bodies"[MeSH Terms] OR ("Lewy"[All Fields] AND "bodies"[All Fields]) OR "lewy bodies"[All Fields]) AND "of parkinson disease"[Title/Abstract]) OR (("lewy bodies"[MeSH Terms] OR ("Lewy"[All Fields] AND "bodies"[All Fields]) OR "lewy bodies"[All Fields]) AND "of parkinson s disease"[Title/Abstract]) OR (("lewy bodies"[MeSH Terms] OR ("Lewy"[All Fields] AND "bodies"[All Fields]) OR "lewy bodies"[All Fields]) AND "of parkinsons disease"[Title/Abstract]) OR (("lewy bodies"[MeSH Terms] OR ("Lewy"[All Fields] AND "bodies"[All Fields]) OR "lewy bodies"[All Fields] OR ("Lewy"[All Fields] AND "Body"[All Fields]) OR "lewy body"[All Fields]) AND "parkinsons disease"[Title/Abstract]) OR "parkinson dementia complex"[Title/Abstract] OR "parkinsons disease"[Title/Abstract])) AND ("Telemedicine"[MeSH Terms] OR ("virtual medicine"[Title/Abstract] OR "medicine virtual"[Title/Abstract] OR "Tele-Referral"[Title/Abstract] OR "Tele-Referral"[Title/Abstract] OR "Tele-Referrals"[Title/Abstract] OR "mobile health"[Title/Abstract] OR "health mobile"[Title/Abstract] OR "mHealth"[Title/Abstract] OR "Telehealth"[Title/Abstract] OR "eHealth"[Title/Abstract] OR "tele intensive care"[Title/Abstract] OR "tele intensive care"[Title/Abstract] OR "Tele-ICU"[Title/Abstract] OR "Tele-ICU"[Title/Abstract] OR "Telecare"[Title/Abstract] OR "Tele-Care"[Title/Abstract] OR "Tele-Care"[Title/Abstract] OR "tele medicine"[Title/Abstract])) AND ("Randomized Controlled Trial"[Publication Type] OR ("controlled trial randomized"[Title/Abstract] OR "randomised controlled study"[Title/Abstract] OR "randomised controlled trial"[Title/Abstract] OR "randomized controlled study"[Title/Abstract] OR "trial randomized controlled"[Title/Abstract] OR "RCT"[Title/Abstract] OR "RCTs"[Title/Abstract]))

**Scopus**

#1

( TITLE-ABS-KEY ( "randomized controlled trial" ) OR TITLE-ABS-KEY ( "controlled trial, randomized" ) OR TITLE-ABS-KEY ( "randomised controlled study" ) OR TITLE-ABS-KEY ( "randomised controlled trial" ) OR TITLE-ABS-KEY ( "randomized controlled study" ) OR TITLE-ABS-KEY ( "trial, randomized controlled" ) OR TITLE-ABS-KEY ( "rct" ) OR TITLE-ABS-KEY ( "rcts" ) )

#2

( TITLE-ABS-KEY ( "telemedicine" ) OR TITLE-ABS-KEY ( "virtual medicine" ) OR TITLE-ABS-KEY ( "medicine, virtual" ) OR TITLE-ABS-KEY ( "tele-referral" ) OR TITLE-ABS-KEY ( "tele referral" ) OR TITLE-ABS-KEY ( "tele-referrals" ) OR TITLE-ABS-KEY ( "mobile health" ) OR TITLE-ABS-KEY ( "health, mobile" ) OR TITLE-ABS-KEY ( "mhealth" ) OR TITLE-ABS-KEY ( "telehealth" ) OR TITLE-ABS-KEY ( "ehealth" ) OR TITLE-ABS-KEY ( "tele-intensive care" ) OR TITLE-ABS-KEY ( "tele intensive care" ) OR TITLE-ABS-KEY ( "tele-icu" ) OR TITLE-ABS-KEY ( "tele icu" ) OR TITLE-ABS-KEY ( "telecare" ) OR TITLE-ABS-KEY ( "tele-care" ) OR TITLE-ABS-KEY ( "tele care" ) OR TITLE-ABS-KEY ( "tele medicine" ) )

#3

( TITLE-ABS-KEY ( "parkinson disease" ) OR TITLE-ABS-KEY ( "parkinsons disease" ) OR TITLE-ABS-KEY ( "idiopathic parkinson's disease" ) OR TITLE-ABS-KEY ( "lewy body parkinson's disease" ) OR TITLE-ABS-KEY ( "parkinson's disease, idiopathic" ) OR TITLE-ABS-KEY ( "parkinson's disease, lewy body" ) OR TITLE-ABS-KEY ( "paralysis agitans" ) OR TITLE-ABS-KEY ( "parkinson's disease" ) OR TITLE-ABS-KEY ( "idiopathic parkinson disease" ) OR TITLE-ABS-KEY ( "lewy body parkinson disease" ) OR TITLE-ABS-KEY ( "primary parkinsonism" ) OR TITLE-ABS-KEY ( "parkinsonism, primary" ) OR TITLE-ABS-KEY ( "parkinson disease, idiopathic" ) OR TITLE-ABS-KEY ( "parkinson's disease, idiopathic" ) OR TITLE-ABS-KEY ( "idiopathic parkinsonism" ) OR TITLE-ABS-KEY ( "lewy bodies of parkinson disease" ) OR TITLE-ABS-KEY ( "lewy bodies of parkinson's disease" ) OR TITLE-ABS-KEY ( "lewy bodies of parkinsons disease" ) OR TITLE-ABS-KEY ( "lewy body parkinsons disease" ) OR TITLE-ABS-KEY ( "parkinson dementia complex" ) OR TITLE-ABS-KEY ( "parkinsons disease" ) )

#3 AND #2 AND #1

( ( TITLE-ABS-KEY ( "parkinson disease" ) OR TITLE-ABS-KEY ( "parkinsons disease" ) OR TITLE-ABS-KEY ( "idiopathic parkinson's disease" ) OR TITLE-ABS-KEY ( "lewy body parkinson's disease" ) OR TITLE-ABS-KEY ( "parkinson's disease, idiopathic" ) OR TITLE-ABS-KEY ( "parkinson's disease, lewy body" ) OR TITLE-ABS-KEY ( "paralysis agitans" ) OR TITLE-ABS-KEY ( "parkinson's disease" ) OR TITLE-ABS-KEY ( "idiopathic parkinson disease" ) OR TITLE-ABS-KEY ( "lewy body parkinson disease" ) OR TITLE-ABS-KEY ( "primary parkinsonism" ) OR TITLE-ABS-KEY ( "parkinsonism, primary" ) OR TITLE-ABS-KEY ( "parkinson disease, idiopathic" ) OR TITLE-ABS-KEY ( "parkinson's disease, idiopathic" ) OR TITLE-ABS-KEY ( "idiopathic parkinsonism" ) OR TITLE-ABS-KEY ( "lewy bodies of parkinson disease" ) OR TITLE-ABS-KEY ( "lewy bodies of parkinson's disease" ) OR TITLE-ABS-KEY ( "lewy bodies of parkinsons disease" ) OR TITLE-ABS-KEY ( "lewy body parkinsons disease" ) OR TITLE-ABS-KEY ( "parkinson dementia complex" ) OR TITLE-ABS-KEY ( "parkinsons disease" ) ) ) AND ( ( TITLE-ABS-KEY ( "telemedicine" ) OR TITLE-ABS-KEY ( "virtual medicine" ) OR TITLE-ABS-KEY ( "medicine, virtual" ) OR TITLE-ABS-KEY ( "tele-referral" ) OR TITLE-ABS-KEY ( "tele referral" ) OR TITLE-ABS-KEY ( "tele-referrals" ) OR TITLE-ABS-KEY ( "mobile health" ) OR TITLE-ABS-KEY ( "health, mobile" ) OR TITLE-ABS-KEY ( "mhealth" ) OR TITLE-ABS-KEY ( "telehealth" ) OR TITLE-ABS-KEY ( "ehealth" ) OR TITLE-ABS-KEY ( "tele-intensive care" ) OR TITLE-ABS-KEY ( "tele intensive care" ) OR TITLE-ABS-KEY ( "tele-icu" ) OR TITLE-ABS-KEY ( "tele icu" ) OR TITLE-ABS-KEY ( "telecare" ) OR TITLE-ABS-KEY ( "tele-care" ) OR TITLE-ABS-KEY ( "tele care" ) OR TITLE-ABS-KEY ( "tele medicine" ) ) ) AND ( ( TITLE-ABS-KEY ( "randomized controlled trial" ) OR TITLE-ABS-KEY ( "controlled trial, randomized" ) OR TITLE-ABS-KEY ( "randomised controlled study" ) OR TITLE-ABS-KEY ( "randomised controlled trial" ) OR TITLE-ABS-KEY ( "randomized controlled study" ) OR TITLE-ABS-KEY ( "trial, randomized controlled" ) OR TITLE-ABS-KEY ( "rct" ) OR TITLE-ABS-KEY ( "rcts" ) ) )

**WOS**

TS=(Parkinson Disease OR Idiopathic Parkinson's Disease OR Lewy Body Parkinson's Disease OR Parkinson's Disease, Idiopathic OR Parkinson's Disease, Lewy Body OR Paralysis Agitans OR Parkinson's Disease OR Idiopathic Parkinson Disease OR Lewy Body Parkinson Disease OR Primary Parkinsonism OR Parkinsonism, Primary OR Parkinson Disease, Idiopathic OR Parkinson's Disease, Idiopathic OR idiopathic parkinsonism OR Lewy bodies of Parkinson disease OR Lewy bodies of Parkinson's disease OR Lewy bodies of Parkinsons disease OR Lewy body Parkinsons disease OR Parkinson dementia complex OR Parkinsons disease) and Preprint Citation Index (Exclude – Database)

TS=(Telemedicine OR Virtual Medicine OR Medicine, Virtual OR Tele-Referral OR Tele Referral OR Tele-Referrals OR Mobile Health OR Health, Mobile OR mHealth OR Telehealth OR eHealth OR Tele-Intensive Care OR Tele Intensive Care OR Tele-ICU OR Tele ICU OR Telecare OR Tele-Care OR Tele Care OR tele medicine) and Preprint Citation Index (Exclude – Database)

TS=(randomized controlled trial OR controlled trial, randomized OR randomised controlled study OR randomised controlled trial OR randomized controlled study OR trial, randomized controlled OR RCT OR RCTs) and Preprint Citation Index (Exclude – Database)

#18 AND #23 AND #27 and Preprint Citation Index (Exclude – Database)

**Embase**

#1

'parkinson disease'/exp OR 'parkinson disease' OR (parkinson AND ('disease'/exp OR disease))

#2

'idiopathic parkinson s disease':ab,ti OR 'lewy body parkinson s disease':ab,ti OR 'parkinson s disease, lewy body':ab,ti OR 'paralysis agitans':ab,ti OR 'parkinson s disease':ab,ti OR 'idiopathic parkinson disease':ab,ti OR 'lewy body parkinson disease':ab,ti OR 'primary parkinsonism':ab,ti OR 'parkinsonism, primary':ab,ti OR 'parkinson disease, idiopathic':ab,ti OR 'parkinson s disease, idiopathic':ab,ti OR 'idiopathic parkinsonism':ab,ti OR 'lewy bodies of parkinson disease':ab,ti OR 'lewy bodies of parkinson s disease':ab,ti OR 'lewy bodies of parkinsons disease':ab,ti OR 'lewy body parkinsons disease':ab,ti OR 'parkinson dementia complex':ab,ti OR 'parkinsons disease':ab,ti

#3

#1 OR #2

#4

'telemedicine'/exp OR telemedicine

#5

'virtual medicine':ab,ti OR 'medicine, virtual':ab,ti OR 'tele-referral':ab,ti OR 'tele referral':ab,ti OR 'tele-referrals':ab,ti OR 'mobile health':ab,ti OR 'health, mobile':ab,ti OR 'mhealth':ab,ti OR 'telehealth':ab,ti OR 'ehealth':ab,ti OR 'tele-intensive care':ab,ti OR 'tele intensive care':ab,ti OR 'tele-icu':ab,ti OR 'tele icu':ab,ti OR 'telecare':ab,ti OR 'tele-care':ab,ti OR 'tele care':ab,ti OR 'tele medicine':ab,ti

#6

#4 OR #5

#7

'randomized controlled trial'/exp OR 'randomized controlled trial' OR (randomized AND controlled AND ('trial'/exp OR trial))

#8

'controlled trial, randomized':ab,ti OR 'randomised controlled study':ab,ti OR 'randomised controlled trial':ab,ti OR 'randomized controlled study':ab,ti OR 'trial, randomized controlled':ab,ti OR 'rct':ab,ti OR 'rcts':ab,ti

#9

#7 OR #8

#10

#3 AND #6 AND #9

**Cochrane**

Search Name:

Date Run: 18/06/2024 08:21:31

Comment:

ID Search Hits

#1 Parkinson Disease 13696

#2 (Idiopathic Parkinson's Disease):ab,ti,kw OR (Lewy Body Parkinson's Disease):ab,ti,kw OR (Parkinson's Disease, Idiopathic):ab,ti,kw OR (Parkinson's Disease, Lewy Body):ab,ti,kw OR (Paralysis Agitans):ab,ti,kw OR (Parkinson's Disease):ab,ti,kw OR (Idiopathic Parkinson Disease):ab,ti,kw OR (Lewy Body Parkinson Disease):ab,ti,kw OR (Primary Parkinsonism):ab,ti,kw OR (Parkinsonism, Primary):ab,ti,kw OR (Parkinson Disease, Idiopathic):ab,ti,kw OR (Parkinson's Disease, Idiopathic):ab,ti,kw OR (idiopathic parkinsonism):ab,ti,kw OR (Lewy bodies of Parkinson disease):ab,ti,kw OR (Lewy bodies of Parkinson's disease):ab,ti,kw OR (Lewy bodies of Parkinsons disease):ab,ti,kw OR (Lewy body Parkinsons disease):ab,ti,kw OR (Parkinson dementia complex):ab,ti,kw OR (Parkinsons disease):ab,ti,kw 13322

#3 #1 OR #2 13906

#4 Telemedicine 6990

#5 (Virtual Medicine):ab,ti,kw OR (Medicine, Virtual):ab,ti,kw OR (Tele-Referral):ab,ti,kw OR (Tele Referral):ab,ti,kw OR (Tele-Referrals):ab,ti,kw OR (Mobile Health):ab,ti,kw OR (Health, Mobile):ab,ti,kw OR (mHealth):ab,ti,kw OR (Telehealth):ab,ti,kw OR (eHealth):ab,ti,kw OR (Tele-Intensive Care):ab,ti,kw OR (Tele Intensive Care):ab,ti,kw OR (Tele-ICU):ab,ti,kw OR (Tele ICU):ab,ti,kw OR (Telecare):ab,ti,kw OR (Tele-Care):ab,ti,kw OR (Tele Care):ab,ti,kw OR (tele medicine):ab,ti,kw 16982

#6 #4 OR #5 21147

#7 randomized controlled trial 1094888

#8 (controlled trial, randomized):ab,ti,kw OR (randomised controlled study):ab,ti,kw OR (randomised controlled trial):ab,ti,kw OR (randomized controlled study):ab,ti,kw OR (trial, randomized controlled):ab,ti,kw OR (RCT):ab,ti,kw OR (RCTs):ab,ti,kw 861352

#9 #7 OR #8 1149968

#10 #3 AND #6 AND #9 171
