# Supplementary material for: Effects of Telehealth Interventions for People With Parkinson Disease: Systematic Review and Meta-Analysis of Randomized Controlled Trials
Source: JMIR Mhealth Uhealth. 2026 Jan 28;14:e70994. doi: 10.2196/70994 (PMC12895161; doi:10.2196/70994)
Supplement: Multimedia Appendix 3 [file mhealth_v14i1e70994_app3.docx]

**R language code**

**#1 Calculate y and V**

>library(metafor)

> Create the Sample Dataset (Replace with Your Actual Data)

>your_data <- data.frame(

Study = c("Deborah G 2016", "Dustin A. Heldman", ...),

n_t = c(15, 9, 26, ...), # Sample size of the experimental group

mean_t = c(21.5, 17.6, 11.4, ...), # Mean of the experimental group

sd_t = c(10.5, 7.11, 6.4, ...), # Standard deviation of the experimental group

n_c = c(16, 9, 24, ...), # Sample size of the control group

mean_c = c(21.2, 22.3, 11.9, ...), # Mean of the control group

sd_c = c(12.8, 8.44, 6.0, ...) # Standard deviation of the control group

)

**#2 The effect size (Hedges 'g) and variance were calculated**

>your_data <- escalc(

measure = "SMD”, # Standardized mean difference

m1i = mean_t, sd1i = sd_t, n1i = n_t,

m2i = mean_c, sd2i = sd_c, n2i = n_c,

data = your_data

)

# Review the Results

head(your_data[, c("yi", "vi")]) # yi=g效应量, vi=方差

**#3 Overall EFFECT ANALYSIS**

Importing csv files

> View(your_data)

> setwd("D:/C_desktop")

> library(Matrix)

>library(metafor)

>dataset <- read.csv("06.csv")

> str(dataset)

# Load the necessary packages

>library(metafor)

# Create unique effector IDs (to handle multiple effectors within the same study)

>dataset$es_id <- paste(dataset$studyID, dataset$effectsizeID, sep = "_")

# Fitting a three-level random effects modelmodel <- rma.mv(

yi = y, # effect size

V = v, # variance of an effect size

random = list(~ 1 | studyID, ~ 1 | es_id), # two-level random effect

data = dataset,

method = "REML", # Restricted great likelihood estimation

test = "t" # Use t-tests (smaller samples are more robust)

)

# View model results

>summary(model)

**#4 Extraction of results**

if (length(model$beta) > 0 && length(model$se) > 0) {

results <- data.frame(

Estimate = model$beta[1],

SE = model$se,

CI_lower = model$ci.lb,

CI_upper = model$ci.ub,

p_value = model$pval,

Tau2_study = if (length(model$sigma2) >= 1) model$sigma2[1] else NA,

Tau2_effect = if (length(model$sigma2) >= 2) model$sigma2[2] else 0

)

**#5 Print formatting results**

cat("\n=== Results of multilevel Meta-analysis ===\n")

cat(sprintf("Overall average effect size: %.4f\n", results$Estimate))

cat(sprintf("standard error: %.4f\n", results$SE))

cat(sprintf("95% confidence interval: [%.4f, %.4f]\n", results$CI_lower, results$CI_upper))

cat(sprintf("p-value: %.4f\n", results$p_value))

cat(sprintf("Inter-study variance (Tau²): %.4f\n", results$Tau2_study))

cat(sprintf("Within-study variance (Tau²): %.4f\n", results$Tau2_effect))

} else {

**#6 Direct manual extraction of results**

cat("\n=== Direct interpretation based on the results you provide ===\n")

cat("Overall average effect size: -0.4237\n")

cat("standard error: 0.2101\n")

cat("95% confidence interval: [-0.8816, 0.0341]\n")

cat("p-value: 0.0667\n")

cat("Inter-study variance (Tau²): 0.2584\n")

cat("Within-study variance (Tau²): 0.0000\n")

}

**#7 I^2^ calculations**

# Define heterogeneity test parameters (from overall effect size output)

Q <- ? # Heterogeneity test Q-value

df <- ? # degrees of freedom

k <- ? # Number of effectors (number of studies)

Calculating the I² statistic

I^2^ <- max(0, (Q - df) / Q) * 100

# Calculate heterogeneity p-value (chi-square test)

p_value <- 1 - pchisq(Q, df)

# Creating a results dataframe

results <- data.frame(

Statistic = c("Q", "df", "k", "I²", "p-value"),

Value = c(Q, df, k, round(I2, 1), round(p_value, 4)),

Interpretation = c(

"Cochran's Q statistic",

"Degrees of freedom",

"Number of effect sizes",

"Total heterogeneity (low if <50%)",

"Significance of heterogeneity"

)

)

# Print calculation results

cat("QOL-2 Heterogeneity Analysis\n")

cat("=============================\n")

print(results, row.names = FALSE)

# Adding a heterogeneity level explanation

cat("\nInterpretation:\n")

if(I2 < 30) {

cat(sprintf("- I² = %.1f%% indicates low heterogeneity\n", I2))

} else if(I2 < 60) {

cat(sprintf("- I² = %.1f%% indicates moderate heterogeneity\n", I2))

} else {

cat(sprintf("- I² = %.1f%% indicates substantial heterogeneity\n", I2))

}

if(p_value > 0.05) {

cat("- p > 0.05 suggests no statistically significant heterogeneity")

} else {

cat("- p ≤ 0.05 suggests statistically significant heterogeneity")

}

forest plot

# Visualization results

# forest plot

forest(model,

slab = meta_dataset$outcomeID,

header = " Results of the study ",

xlab = " effect size ",

cex = 0.8,

mlab = " Overall average effect size ")

**#8 Subgroup analysis by follow-up time**

# The duration of follow-up was categorized into short-term (<3 months) and long-term

meta_dataset$followup_group <- ifelse(meta_dataset$Follow_up_duration < 3, " short-term ", " long term ")

# cluster analysis model

model_followup <- rma.mv(

yi = y,

V = v,

random = list(~ 1 | studyID, ~ 1 | es_id),

mods = ~ followup_group,

data = meta_dataset,

method = "REML"

)

# View Results

summary(model_followup)

**#9 Subgroup analysis by intervention group**

# Creating Intervention Type Variables

meta_dataset$intervention_type <- ifelse(meta_dataset$Digital == 1, " Digital interventions ", ifelse(meta_dataset$Telephone == 1, " Telephone interventions ", "other"))

# cluster analysis model

model_intervention <- rma.mv(

yi = y,

V = v,

random = list(~ 1 | studyID, ~ 1 | es_id),

mods = ~ intervention_type,

data = meta_dataset,

method = "REML"

)

# View Results

summary(model_intervention)

# Visualize grouped results

forest(model_intervention,

slab = meta_dataset$outcomeID,

header = " Grouping by type of intervention ",

xlab = " effect size ",

cex = 0.7,

mlab = " overall effect ")

Follow-up time as a continuous variable

**#10 Analysis of continuous moderator variables**

model_duration <- rma.mv(

yi = y,

V = v,

random = list(~ 1 | studyID, ~ 1 | es_id),

mods = ~ Follow_up_duration,

data = meta_dataset,

method = "REML"

)

# View Results

summary(model_duration)

**#11 Visualizing the dose-response relationship**

plot(dataset$Follow_up_duration, dataset$y,

xlab = "Follow-up period (months)", ylab = "Effect size",

pch = 19, col = "blue", cex = 1.5/sqrt(dataset$v))

abline(model_duration, lwd = 2, col = "red")

title("The relationship between effect size and follow-up time")

**#12 Publication bias test**

funnel plot

# basic funnel plot

funnel(base_model, main = "Funnel Plot")

# Enhanced Funnel plot

funnel(base_model, yaxis = "seinv", level = c(90, 95, 99),

shade = c("white", "gray75", "gray55"),

refline = 0, legend = TRUE, main = "Funnel Plot")

Egger's regression test

# standard error of calculation

meta_dataset$se <- sqrt(meta_dataset$v)

# Egger's test

egger_test <- rma.mv(

yi = y,

V = v,

random = list(~ 1 | studyID, ~ 1 | es_id),

mods = ~ se, # Adding standard errors as predictor

data = meta_dataset,

method = "REML"

)# View Results

summary(egger_test)

**#13 sensitivity analysis**

(restarting the new work interface)

Re-import the file csv.

library(ggplot2)

library(metafor)

**#** Fitting a three-level random effects model

model <- rma.mv(

yi = y, # effect size

V = v, # variance of an effect size

random = list(~ 1 | studyID, ~ 1 | es_id), # two-level random effect

data = dataset,

method = "REML", # Restricted great likelihood estimation

test = "t" # Use t-tests (smaller samples are more robust)

)

Sensitivity analysis by study (optimal method)

study_ids <- unique(meta_dataset$studyID)

sensitivity_results <- data.frame(

study_removed = character(),

estimate = numeric(),

ci_lb = numeric(),

ci_ub = numeric(),

tau2_study = numeric(),

stringsAsFactors = FALSE

)

# Cycling through the studies one by one to eliminate

for (study_id in study_ids) {

# Creating a dataset that excludes the current study

temp_data <- meta_dataset[meta_dataset$studyID != study_id, ]

# fit model

temp_model <- rma.mv(

yi = y,

V = v,

random = list(~ 1 | studyID, ~ 1 | es_id),

data = temp_data,

method = "REML",

control = list(optimizer = "uobyqa")

)

# Storing results

sensitivity_results <- rbind(sensitivity_results, data.frame(

study_removed = as.character(study_id),

estimate = temp_model$beta[1],

ci_lb = temp_model$ci.lb,

ci_ub = temp_model$ci.ub,

tau2_study = temp_model$sigma2[1]

))

}

# Add full model results

full_model_result <- data.frame(

study_removed = "None",

estimate = base_model$beta[1],

ci_lb = base_model$ci.lb,

ci_ub = base_model$ci.ub,

tau2_study = base_model$sigma2[1]

)

**#14 merger results**

all_results <- rbind(full_model_result, sensitivity_results)

Calculation of percentage change

all_results$change_percent <- (all_results$estimate - model$beta[1]) / model$beta[1] * 100

Visualization Results - Forest plotfor Professional

ggplot(all_results, aes(x = estimate, y = reorder(study_removed, estimate))) +

geom_point(aes(color = ifelse(study_removed == "None", "Full Model", "Study Removed")), size = 3) +

geom_errorbarh(aes(xmin = ci_lb, xmax = ci_ub, height = 0.2),

color = "blue", alpha = 0.7) +

geom_vline(xintercept = model$beta[1], linetype = "dashed", color = "red") +

geom_vline(xintercept = 0, linetype = "dotted", color = "black") +

geom_label(aes(label = sprintf("%.3f (%.1f%%)", estimate, change_percent)),

nudge_y = 0.2, size = 3) +

scale_color_manual(values = c("Full Model" = "red", "Study Removed" = "blue")) +

labs(

title = "Sensitivity analysis: Eliminate the influence on the overall effect size one by one in each study",

subtitle = paste("Base model effect size =", round(model$beta[1], 3),

"95% CI [", round(model$ci.lb, 3), ",",

round(model$ci.ub, 3), "]"),

x = "Overall effect size estimation",

y = "The excluded research ID",

caption = "Label format: Effect size estimate (percentage change compared to the base model)",

color = "Model type"

) +

theme_minimal() +

theme(

plot.title = element_text(hjust = 0.5, face = "bold"),

plot.subtitle = element_text(hjust = 0.5),

legend.position = "bottom"

)

Visualizing Heterogeneous Change

ggplot(all_results, aes(x = study_removed, y = tau2_study)) +

geom_bar(stat = "identity", fill = "steelblue", alpha = 0.7) +

geom_hline(yintercept = model$sigma2[1], linetype = "dashed", color = "red") +

labs(

title = "Variation in inter-study heterogeneity (Tau²)",

x = "The excluded research ID",

y = "Interstudy variance (Tau²)",

caption = "The red dotted line indicates the heterogeneity among research groups of the basic model."

) +

theme_minimal() +

theme(plot.title = element_text(hjust = 0.5))

Identify key research

# Calculate impact index = |change in effect size| + |change in heterogeneity|

all_results$impact_index <- abs(all_results$change_percent) +

abs((all_results$tau2_study - model$sigma2[1]) / model$sigma2[1] * 100)

# Sort and display the most influential studies

most_influential <- all_results[order(-all_results$impact_index), ]

cat("Studies with the greatest impact on results:\n")

print(most_influential[1:min(5, nrow(most_influential)), ])

# Sort and display the most influential studies

most_influential <- all_results[order(-all_results$impact_index), ]

cat("Studies with the greatest impact on results:\n")

print(most_influential[1:min(5, nrow(most_influential)), ])

# 7. Save results

write.csv(all_results, "study_level_sensitivity_analysis.csv", row.names = FALSE)

# 8. Professional Interpretation

cat("\n=== Sensitivity Analysis Professional Interpretation ===\n")

cat("Base model overall effect size:", round(model$beta[1], 4),

"(95% CI:", round(model$ci.lb, 4), "to", round(model$ci.ub, 4), ")\n")

cat("Inter-study heterogeneity (Tau²):", round(model$sigma2[1], 4), "\n\n")

cat("Sensitivity analysis results:\n")

cat("- Range of effect size changes:", round(min(all_results$estimate), 4), "to", round(max(all_results$estimate), 4), "\n")

cat("- Maximum change studies:", most_influential$study_removed[1],

"(change:", round(most_influential$change_percent[1], 1), "%)\n")

cat("- Range of variation in heterogeneity:", round(min(all_results$tau2_study), 4), "to",

round(max(all_results$tau2_study), 4), "\n\n")

cat("Concluding recommendations:\n")

if (all(all_results$ci_lb < 0 & all_results$ci_ub < 0)) {

cat("-All models show significant negative effects \n")

} else if (all(all_results$ci_lb > 0 & all_results$ci_ub > 0)) {

cat("- All models showed significant positive effects \n")

} else {

cat("- Robustness of results: ",

if (max(abs(all_results$change_percent)) < 10) "高" else "中",

"\n")

cat("- The basic conclusions are consistent in terms of direction across the models, " \n")

sum(sign(all_results$estimate) == sign(model$beta[1])),

}

Percentage of total variability for each level of variability

# Calculate the total variation decomposition including sampling error

calculate_total_variance_components <- function(model, dataset) {

# Extracting the random effects variance

tau2_study <- model$sigma2[1]

tau2_effect <- model$sigma2[2]

# Calculate the average sampling error

mean_sampling_variance <- mean(dataset$v)

# Calculate total variance

total_variance <- tau2_study + tau2_effect + mean_sampling_variance

# Calculate the percentage of each component

prop_study <- (tau2_study / total_variance) * 100

prop_effect <- (tau2_effect / total_variance) * 100

prop_sampling <- (mean_sampling_variance / total_variance) * 100

# Creating a results dataframe

variance_df <- data.frame(

Component = c("Between Studies",

"Within Studies (Between Effects)",

"Sampling Error"),

Variance = c(tau2_study, tau2_effect, mean_sampling_variance),

Proportion = c(prop_study, prop_effect, prop_sampling),

Percentage = sprintf("%.1f%%", c(prop_study, prop_effect, prop_sampling))

)

# Add total variance row

total_row <- data.frame(

Component = "Total Variance",

Variance = total_variance,

Proportion = 100,

Percentage = "100.0%"

)

# Merger results

rbind(variance_df, total_row)

}

# Calculate and display results Calculate and display results

total_variance_components <- calculate_total_variance_components(model, dataset)

print(total_variance_components)

# visualization

pie(total_variance_components$Proportion[1:3],

labels = paste(total_variance_components$Component[1:3],

"\n", total_variance_components$Percentage[1:3]),

col = c("skyblue", "lightgreen", "salmon"),

main = "Total Variance Components")
