# Supplementary material for: Effects of Telehealth Interventions for People With Parkinson Disease: Systematic Review and Meta-Analysis of Randomized Controlled Trials
Source: JMIR Mhealth Uhealth. 2026 Jan 28;14:e70994. doi: 10.2196/70994 (PMC12895161; doi:10.2196/70994)

**Figure S1: The three-level meta-analysis of eight studies (13 effect sizes) assessing quality of life with PDQ scales-Overall Effect Size Results**


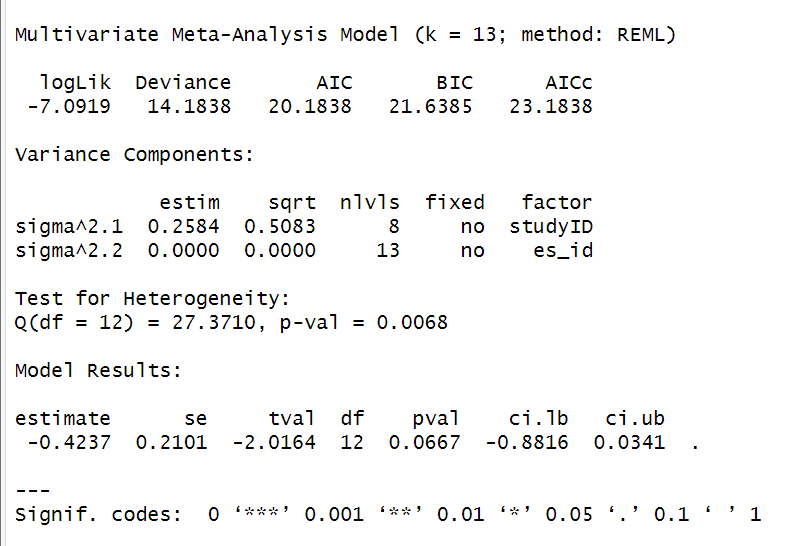


**Figure S2: The three-level meta-analysis of eight studies (13 effect sizes) assessing quality of life with PDQ scales-Total variation**


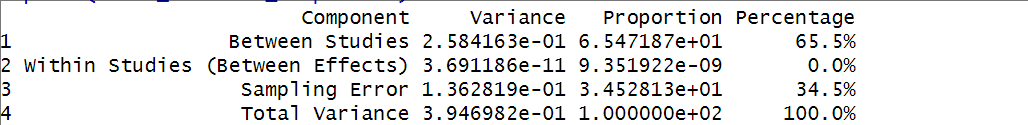


**Figure S3: The three-level meta-analysis of eight studies (13 effect sizes) assessing quality of life with PDQ scales-*I^2^***


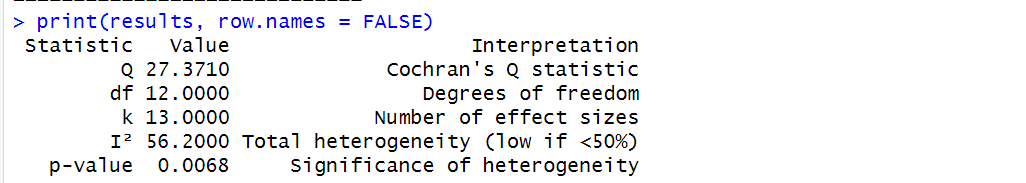


**Figure S4: The three-level meta-analysis of eight studies (13 effect sizes) assessing quality of life with PDQ scales-Subgroup analysis(Intervention Type)**


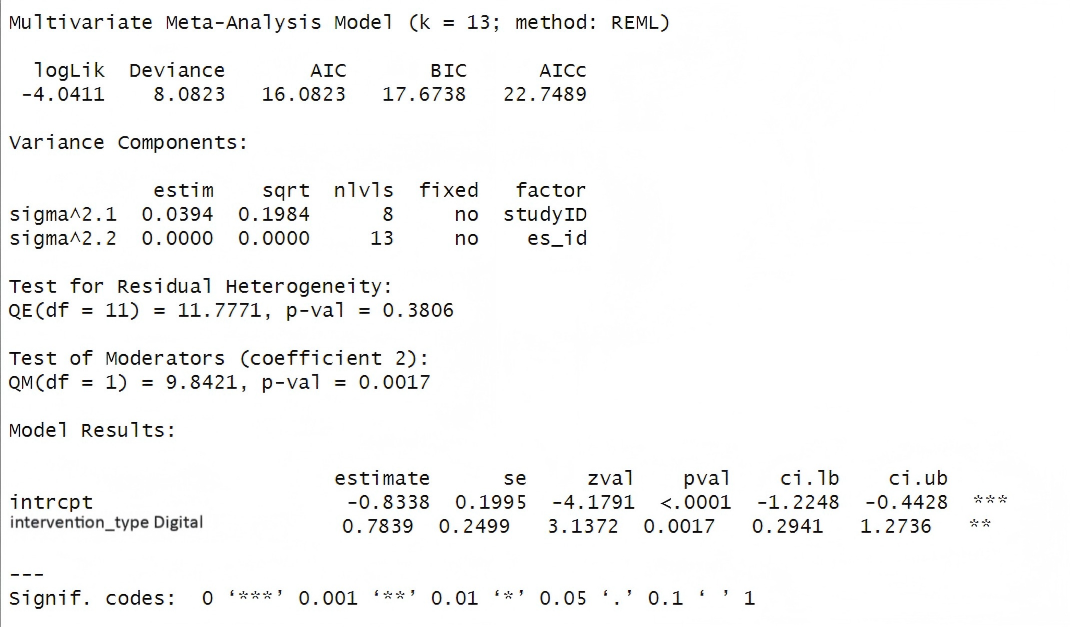


**Figure S5: The three-level meta-analysis of eight studies (13 effect sizes) assessing quality of life with PDQ scales-Subgroup analysis(Follow-up duration_categorical)**


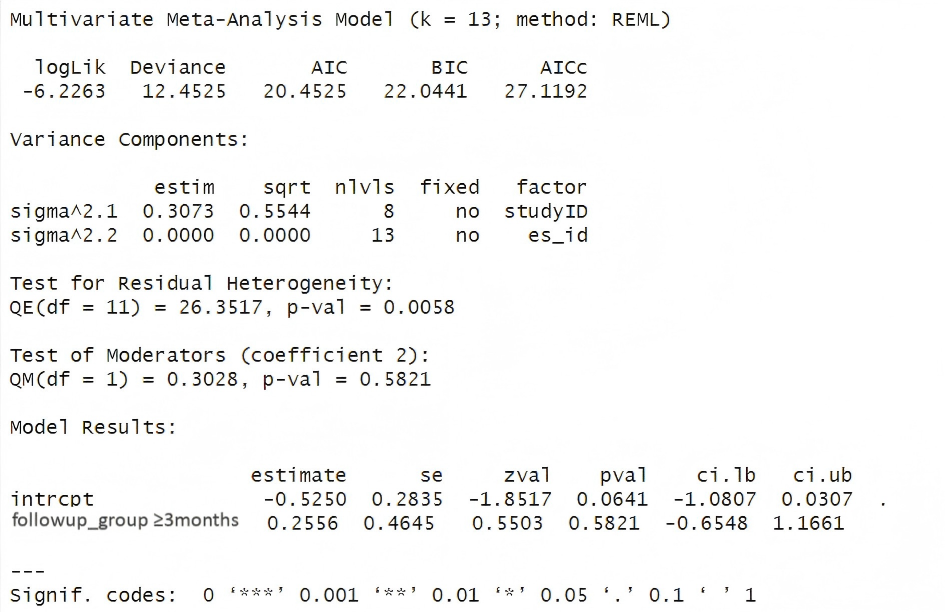


**Figure S6: The three-level meta-analysis of eight studies (13 effect sizes) assessing quality of life with PDQ scales-Subgroup analysis(Follow-up duration_continuous)**

**Figure S7: The three-level meta-analysis of eight studies (13 effect sizes) assessing quality of life with PDQ scales-Subgroup analysis(The relationship between effect size and follow-up time)**


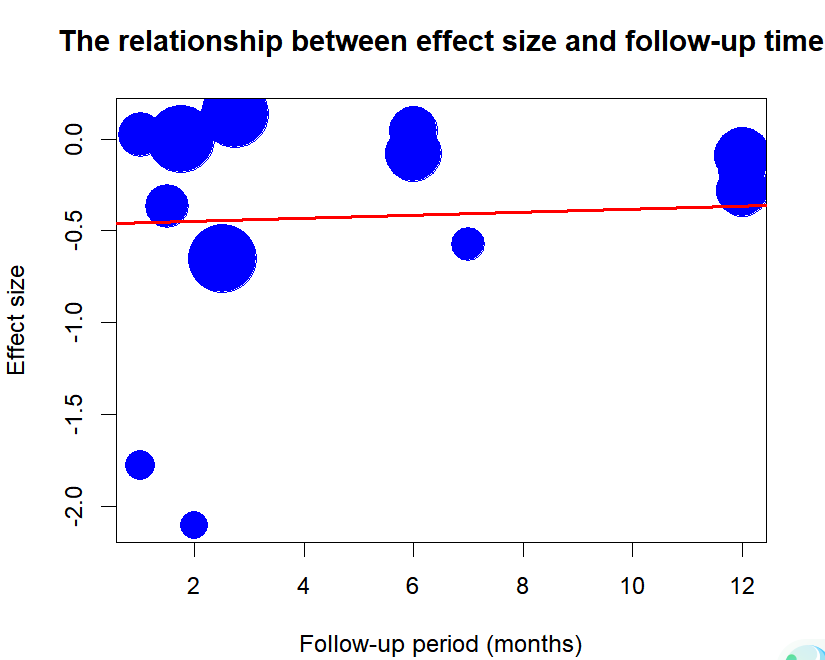


**Figure S8: The three-level meta-analysis of eight studies (13 effect sizes) assessing quality of life with PDQ scales-Sensitivity analysis**


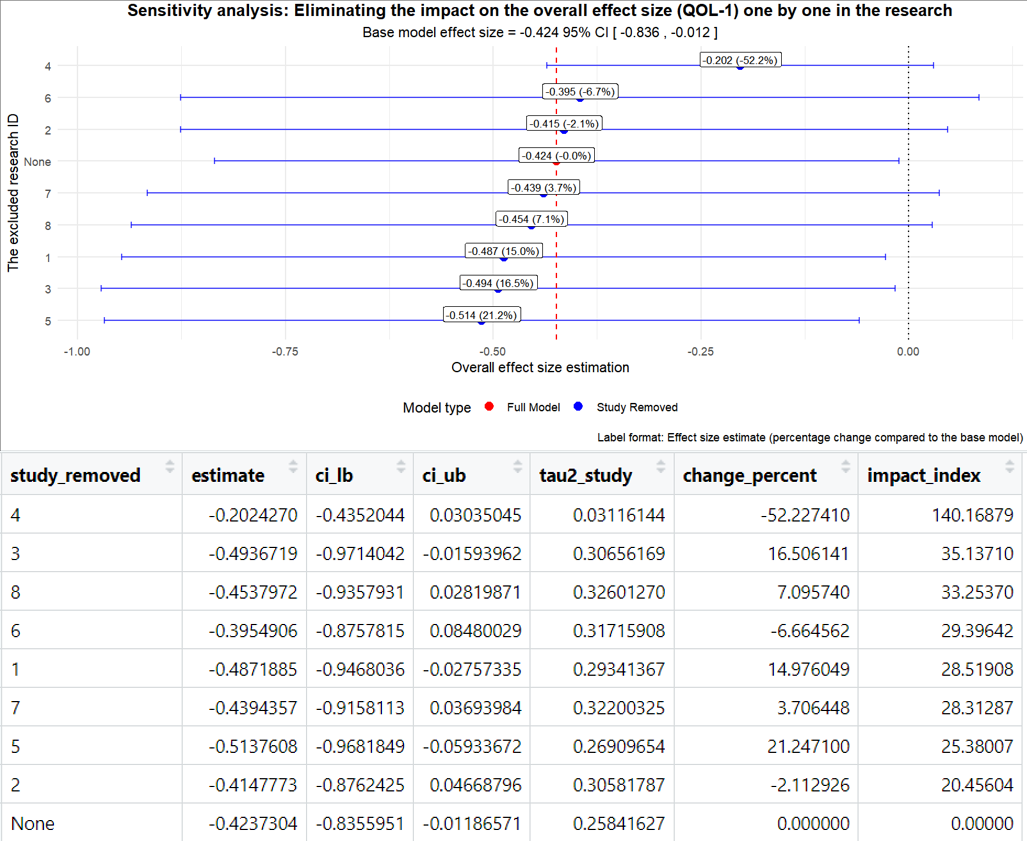


1=Deborah G 2016; 2=Dustin A. Heldman 2017; 3=Jayne R 2016; 4=Pastana Ramos 2023; 5=Marialuisa Gandolfi 2017; 6=Martin Kraepelien 2020; 7=Sefa Eldemir 2023; 8=Terry D. Ellis 2018

**Figure S9: The three-level meta-analysis of eight studies (13 effect sizes) assessing quality of life with PDQ scales-Funnel plot and multilevel Egger's test**


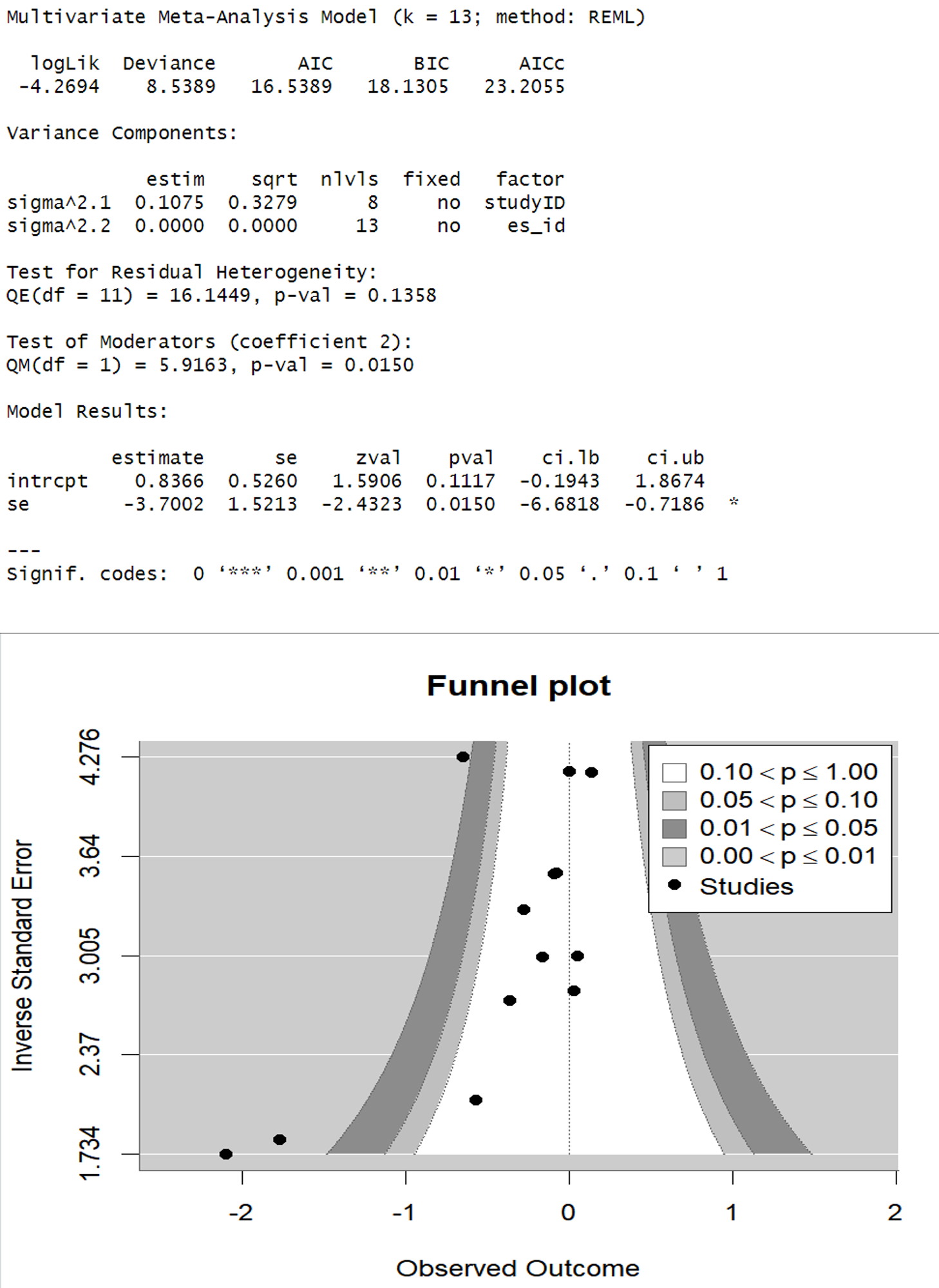


**Figure S10: The three-level meta-analysis of three studies (5 effect sizes) assessing quality of life with SF-36 and BBQ scales-Overall Effect Size Results**


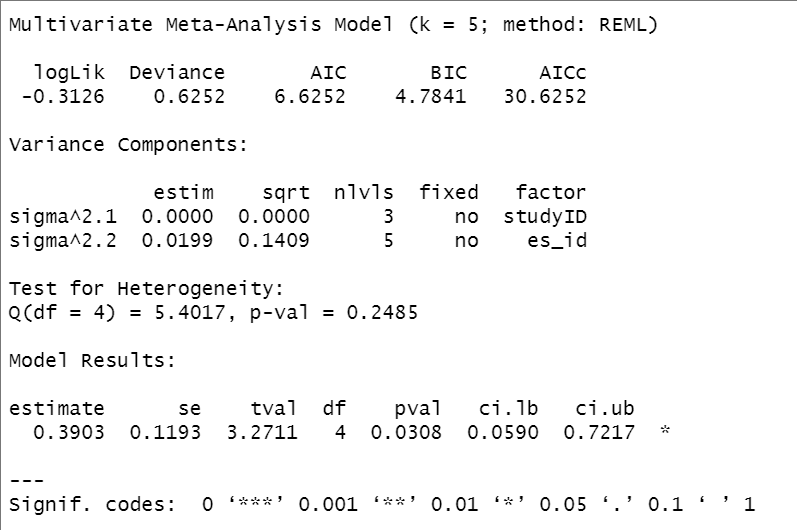


**Figure S11: The three-level meta-analysis of three studies (5 effect sizes) assessing quality of life with SF-36 and BBQ scales-Total variation**


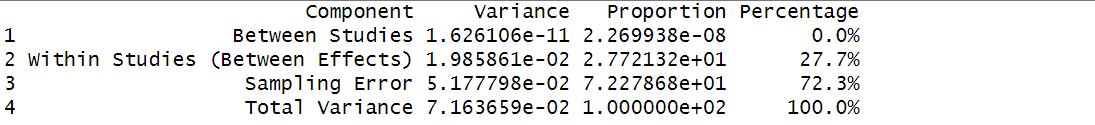


**Figure S12: The three-level meta-analysis of three studies (5 effect sizes) assessing quality of life with SF-36 and BBQ scales-*I^2^***


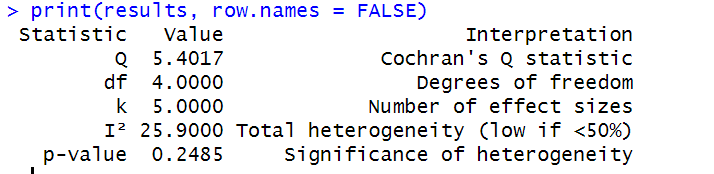


**Figure S13: The three-level meta-analysis of three studies (5 effect sizes) assessing quality of life with SF-36 and BBQ scales-Subgroup analysis(Follow-up duration_categorical)**


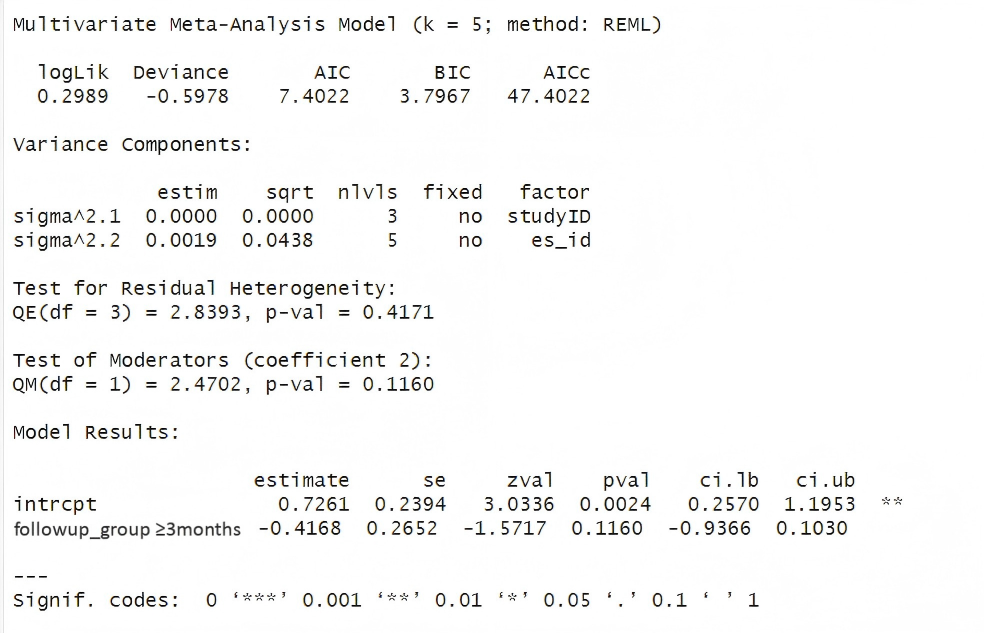


**Figure S14: The three-level meta-analysis of three studies (5 effect sizes) assessing quality of life with SF-36 and BBQ scales-Subgroup analysis(Follow-up duration_continuous)**


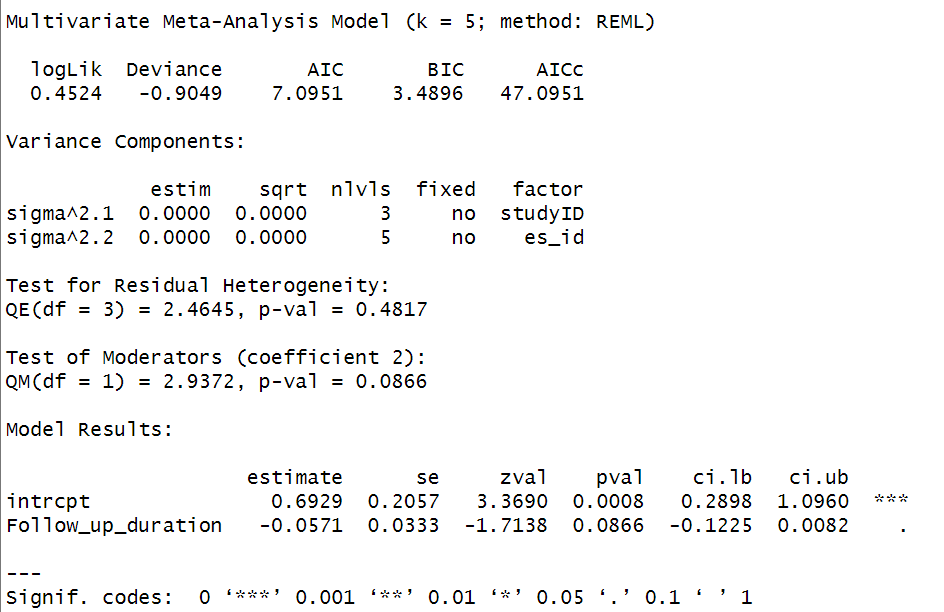


**Figure S15: The three-level meta-analysis of three studies (5 effect sizes) assessing quality of life with SF-36 and BBQ scales-Subgroup analysis(The relationship between effect size and follow-up time)**


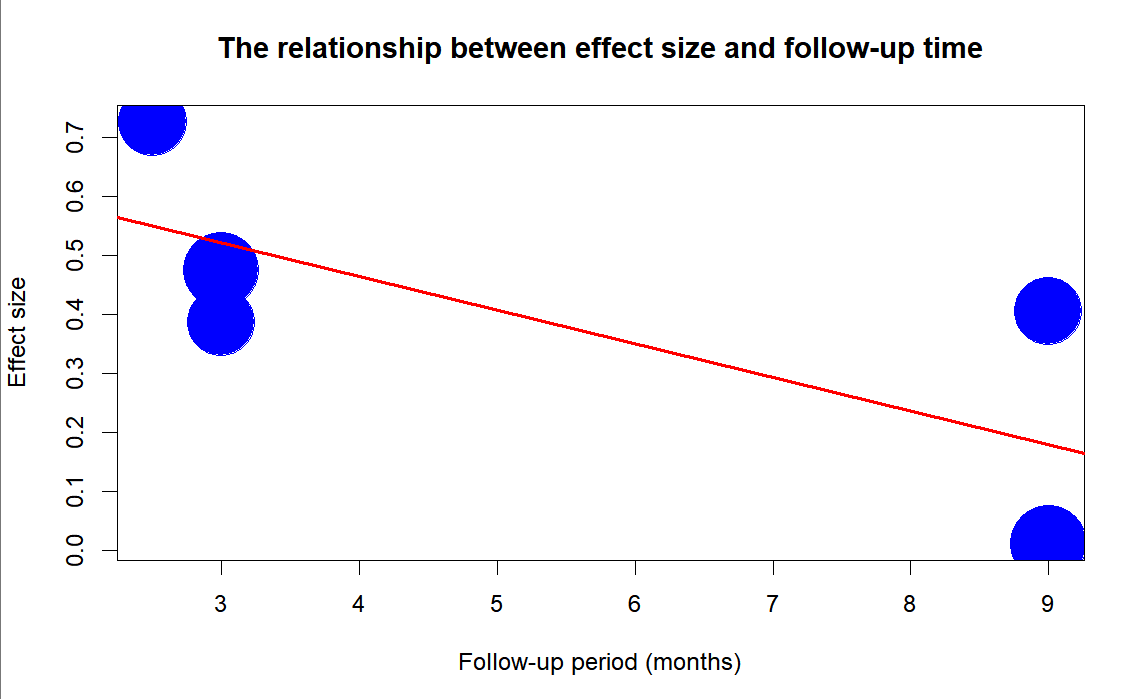


**Figure S16: The three-level meta-analysis of three studies (5 effect sizes) assessing quality of life with SF-36 and BBQ scales-Sensitivity analysis**


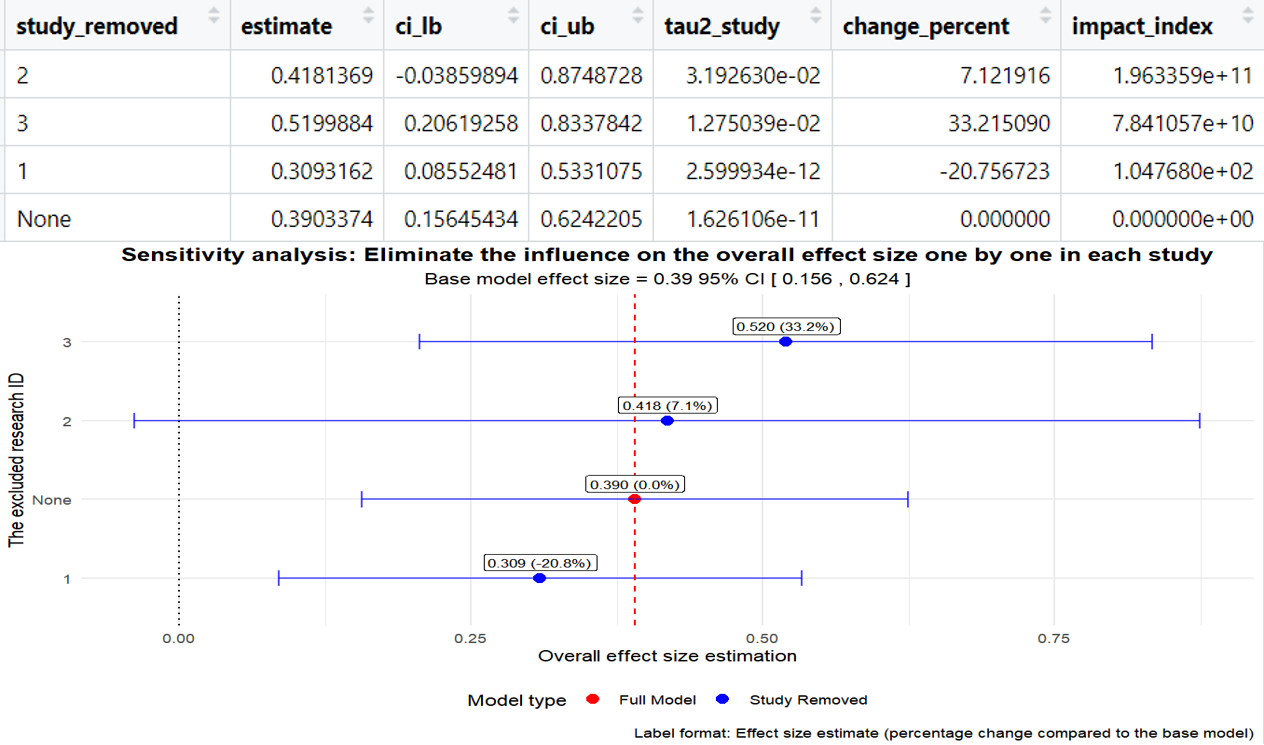


1=Martin Kraepelien 2020; 2=Roseanne D. Dobkin 2020; 3=Roseanne D. Dobkin 2021

**Figure S17: The three-level meta-analysis of three studies (5 effect sizes) assessing quality of life with SF-36 and BBQ scales-Funnel plot and multilevel Egger's test**


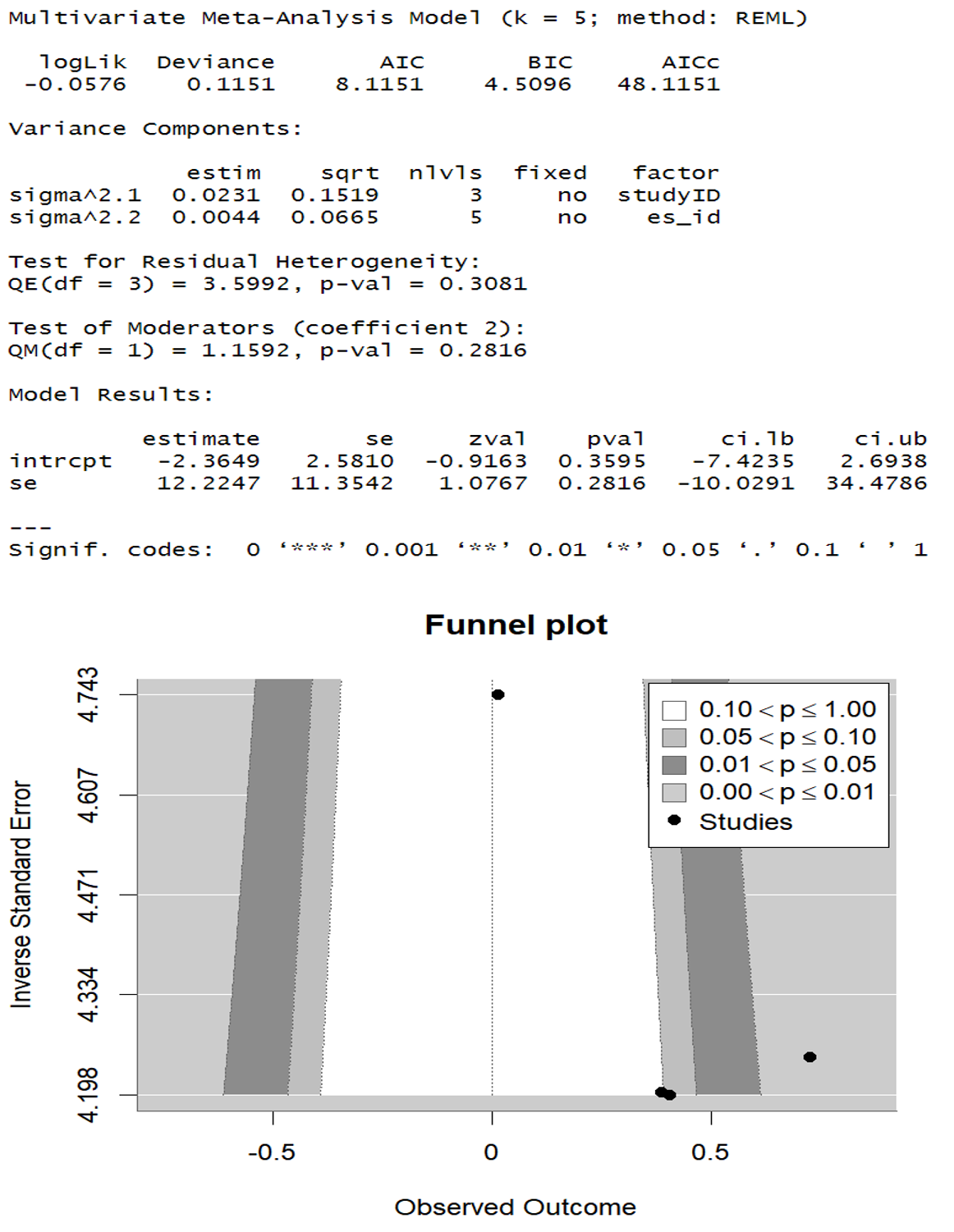


**Figure S17: The three-level meta-analysis of five studies (17 effect sizes) assessing depression -Overall Effect Size Results**

**
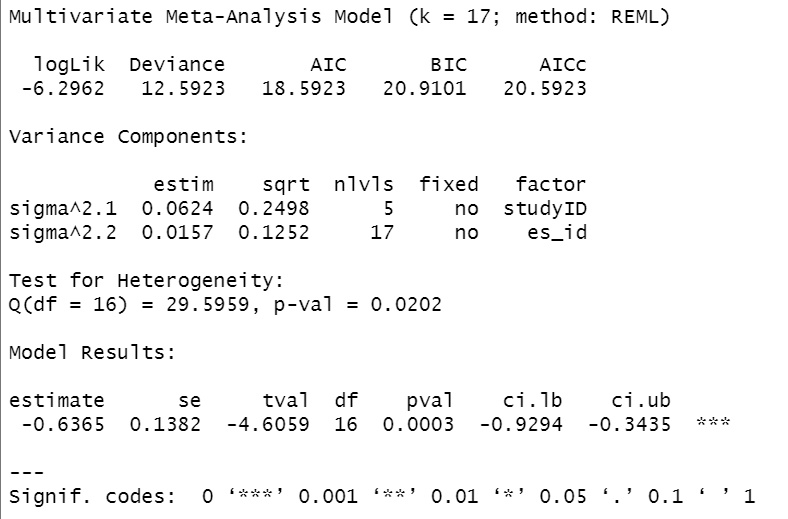
**

**Figure S18: The three-level meta-analysis of five studies (17 effect sizes) assessing depression -Total variation**

**
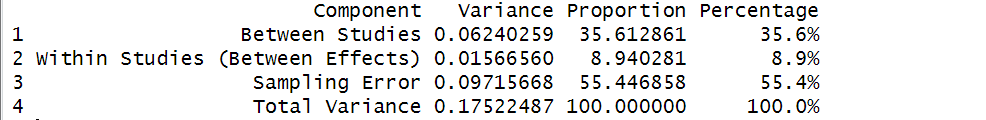
**

**Figure S19: The three-level meta-analysis of five studies (17 effect sizes) assessing depression -*I^2^***

**
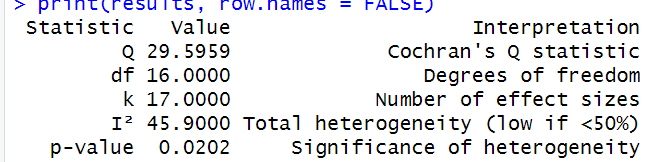
**

**Figure S20: The three-level meta-analysis of five studies (17 effect sizes) assessing depression -Subgroup analysis(Intervention Type)**

**
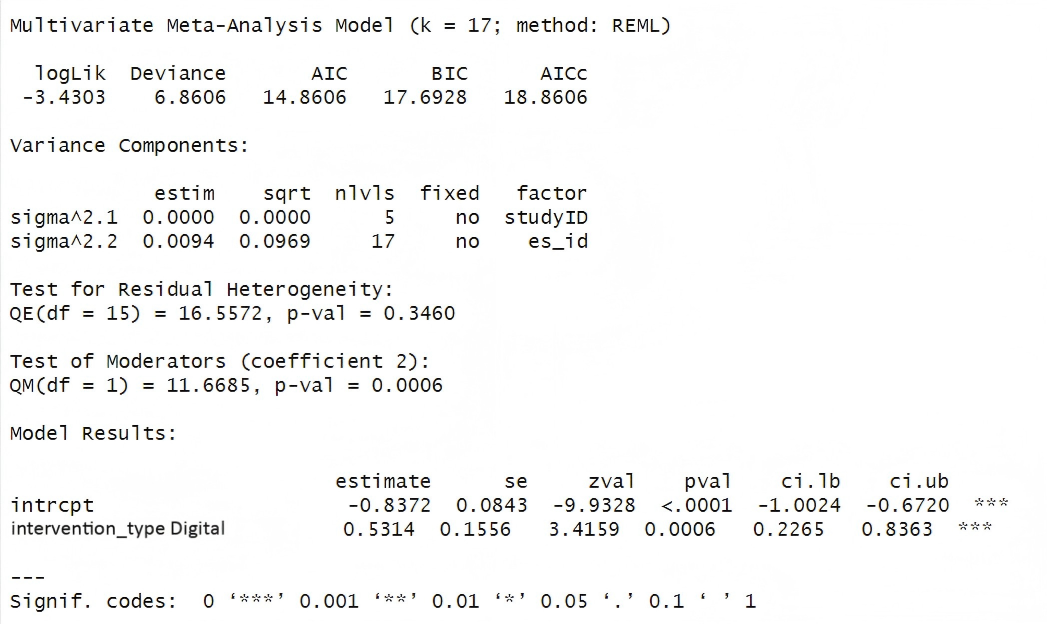
**

**Figure S21: The three-level meta-analysis of five studies (17 effect sizes) assessing depression -Subgroup analysis(Follow-up duration_categorical)**


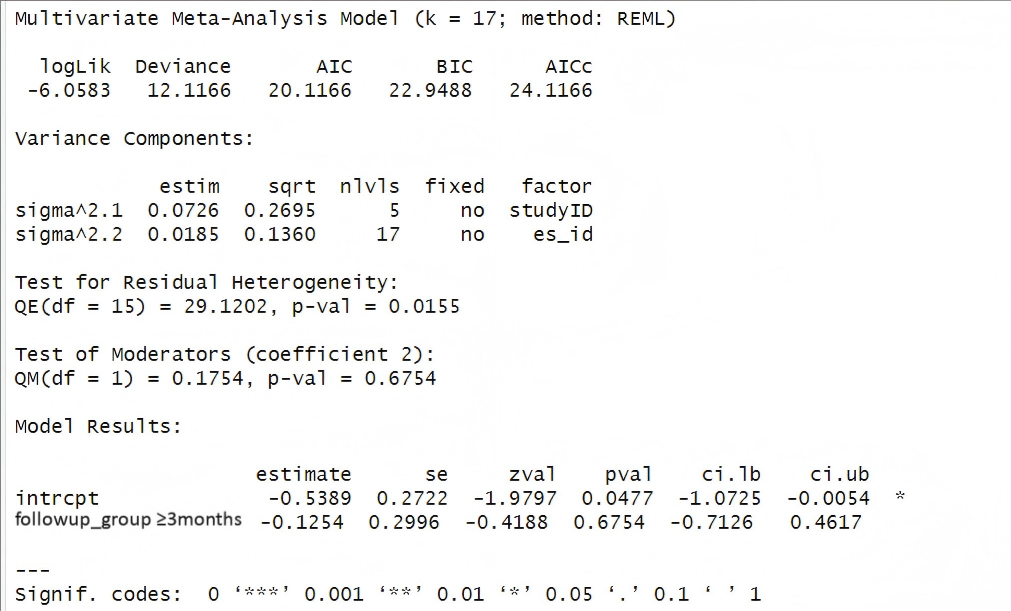


**Figure S22: The three-level meta-analysis of five studies (17 effect sizes) assessing depression -Subgroup analysis(Follow-up duration_continuous)**


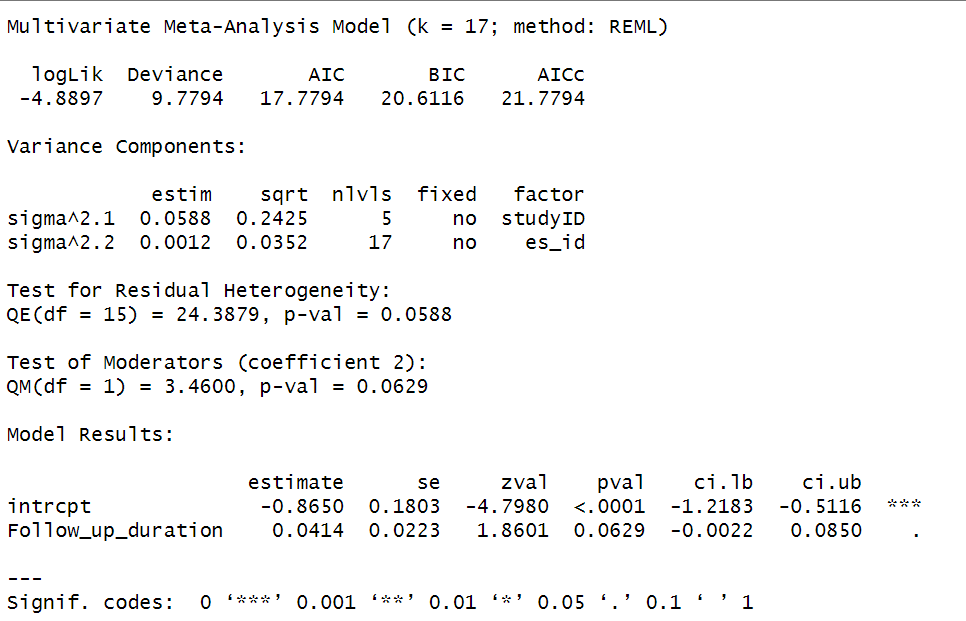


**Figure S23: The three-level meta-analysis of five studies (17 effect sizes) assessing depression -Subgroup analysis(The relationship between effect size and follow-up time)**

**
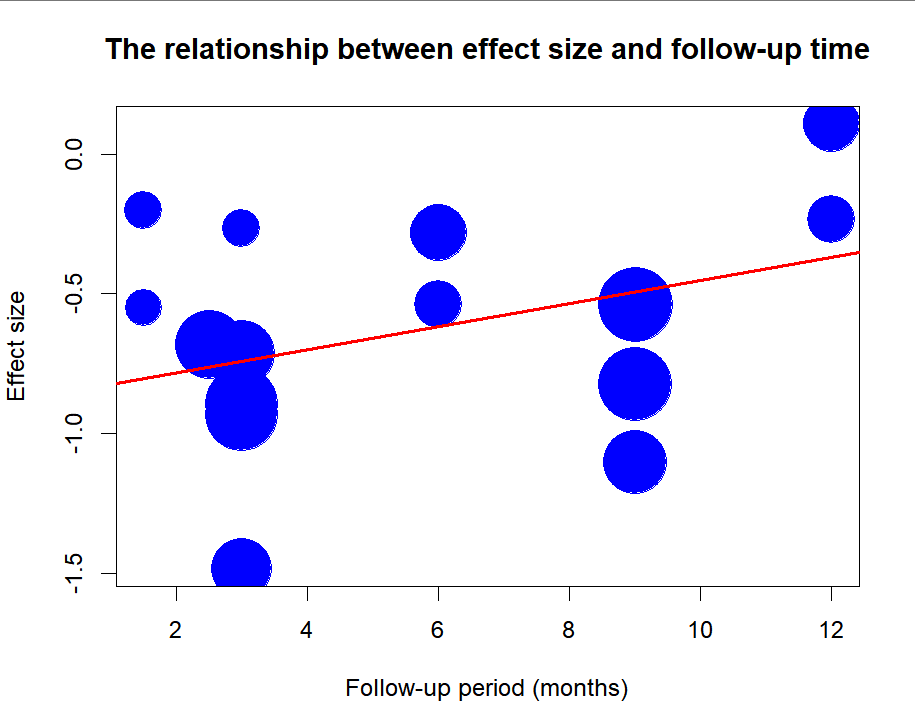
**

**Figure S24: The three-level meta-analysis of five studies (17 effect sizes) assessing depressions-Sensitivity analysis**


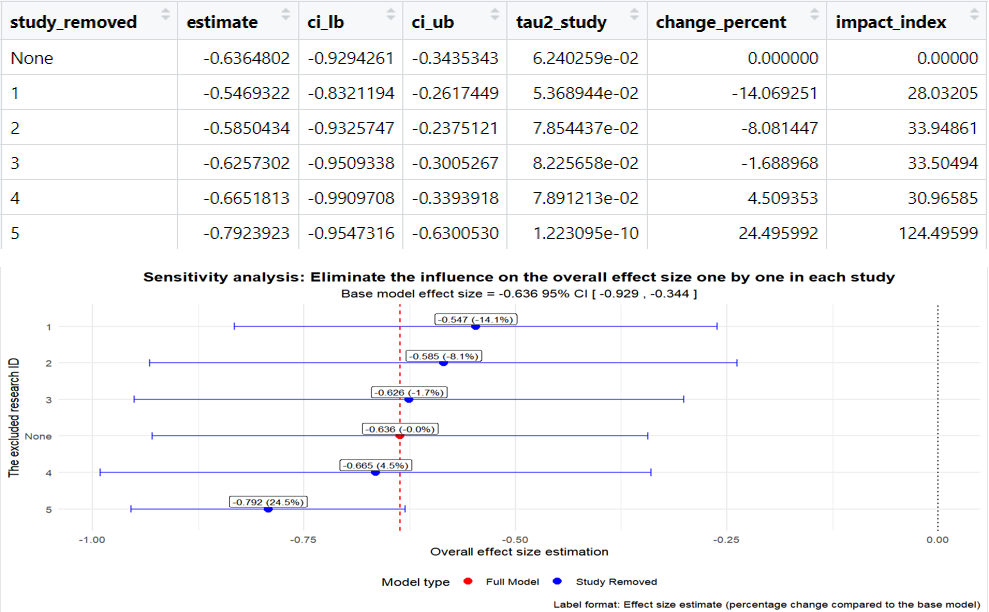


1=Roseanne D. Dobkin 2020; 2=Roseanne D. Dobkin 2021; 3=Martin Kraepelien 2020; 4=Maria Grazia Maggio 2024; 5=Jayne R 2016.

**Figure S25: The three-level meta-analysis of five studies (17 effect sizes) assessing depression -Funnel plot and multilevel Egger's test**


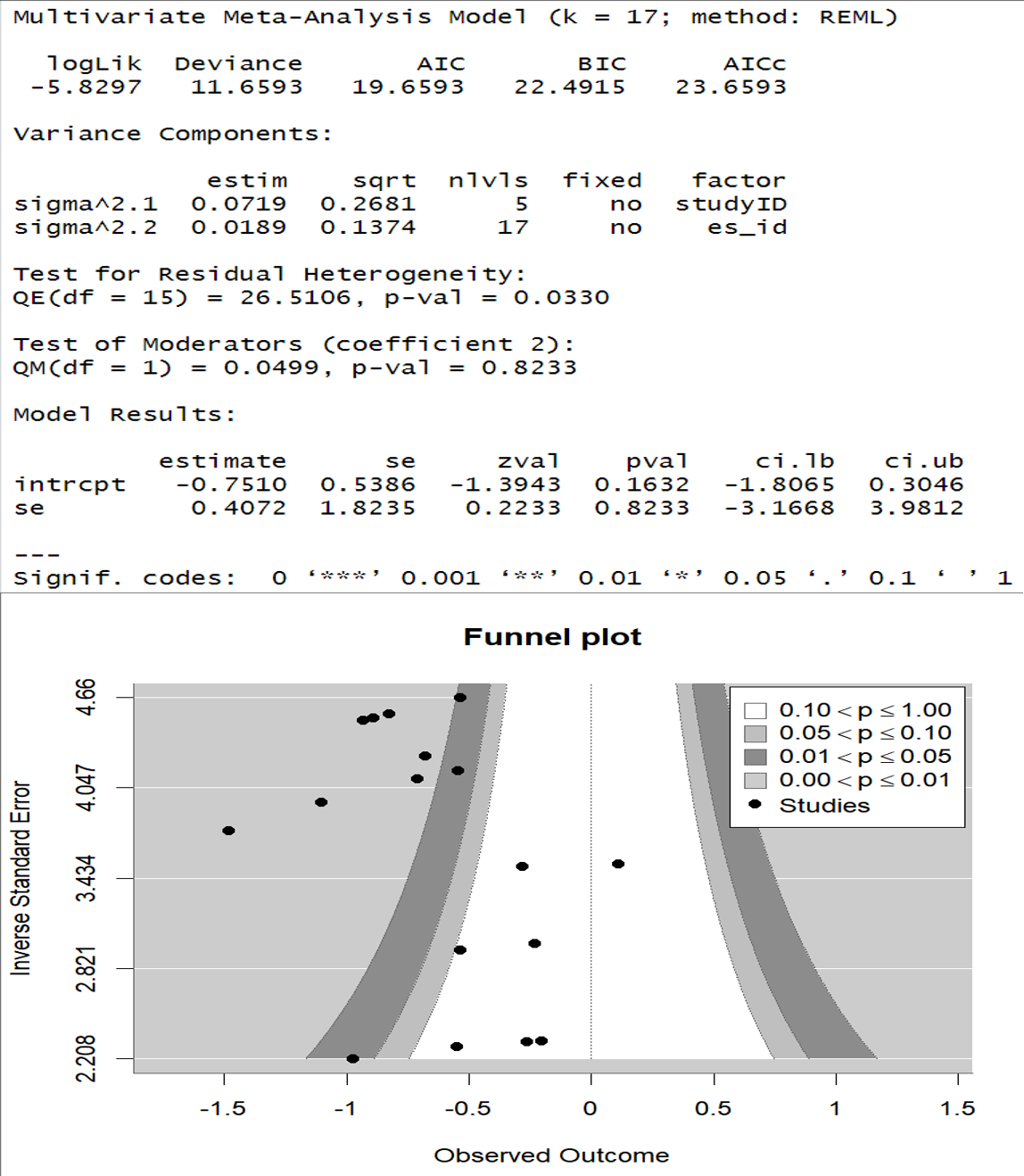


**Figure S26: The three-level meta-analysis of three studies (5 effect sizes) assessing anxiety-Overall Effect Size Results**


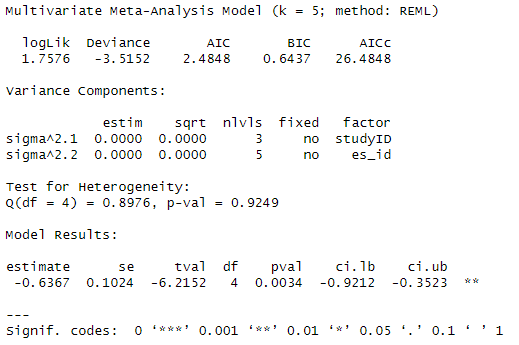


**Figure S27: The three-level meta-analysis of three studies (5 effect sizes) assessing anxiety-Total variation**


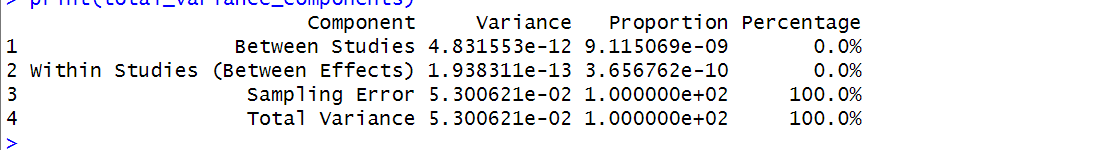


**Figure S28: The three-level meta-analysis of three studies (5 effect sizes) assessing anxiety-*I^2^***


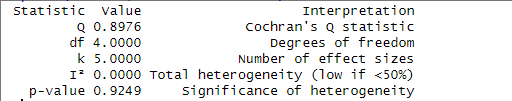


**Figure S29: The three-level meta-analysis of three studies (5 effect sizes) assessing anxiety-Subgroup analysis(Follow-up duration_categorical)**


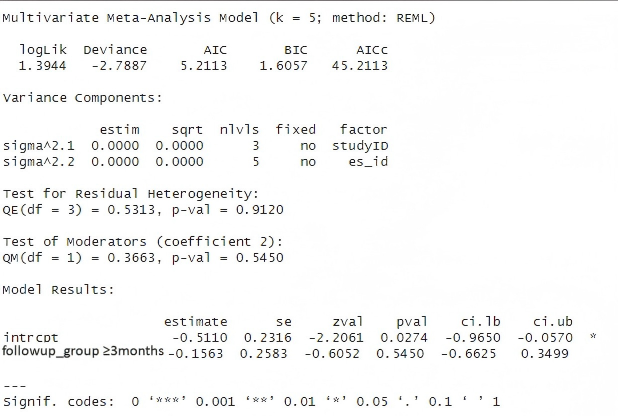


**Figure S30: The three-level meta-analysis of three studies (5 effect sizes) assessing anxiety-Subgroup analysis(Follow-up duration_continuous)**

**
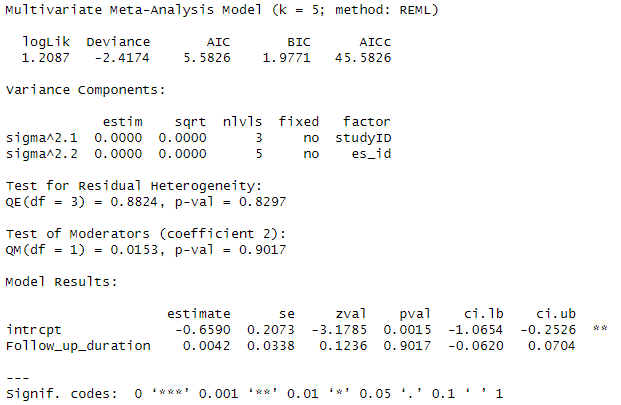
**

**Figure S31: The three-level meta-analysis of three studies (5 effect sizes) assessing anxiety-Subgroup analysis(The relationship between effect size and follow-up time)**


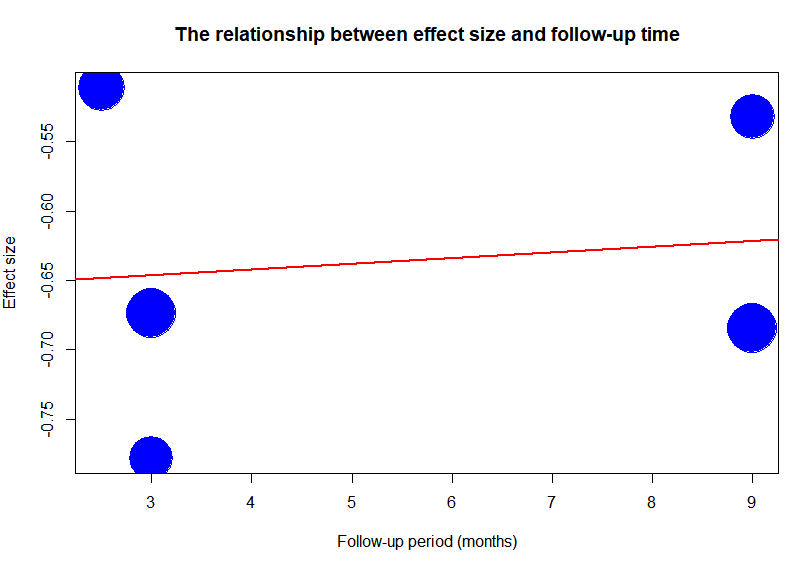


**Figure S32: The three-level meta-analysis of three studies (5 effect sizes) assessing anxiety-Sensitivity analysis**


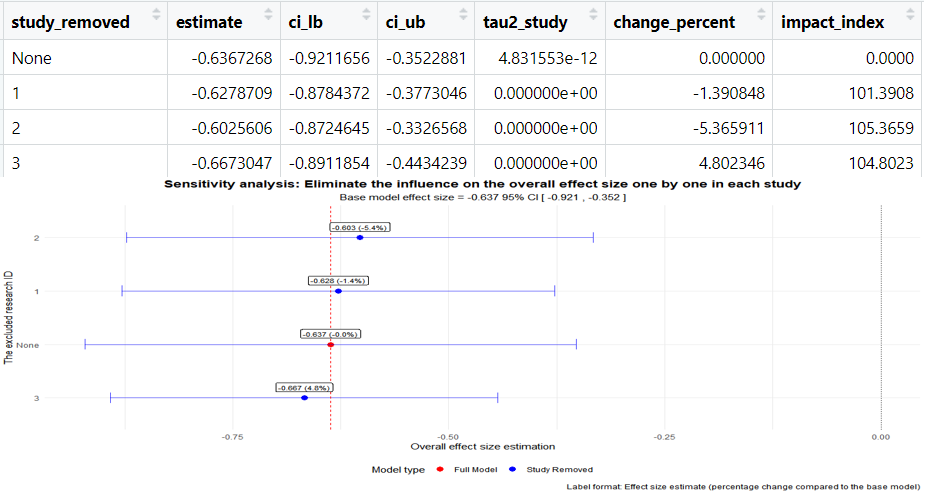


1=Roseanne D. Dobkin 2020; 2=Roseanne D. Dobkin 2021; 3=Martin Kraepelien 2020

**Figure S33: The three-level meta-analysis of three studies (5 effect sizes) assessing anxiety-Funnel plot and multilevel Egger's test**


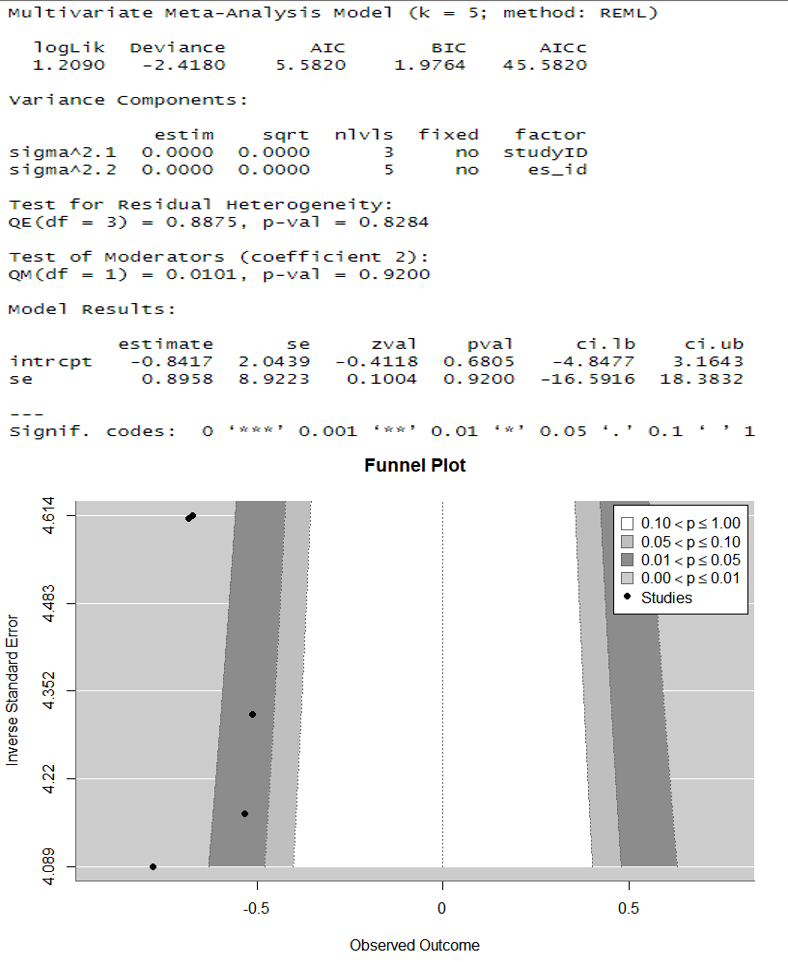


**Figure S34: The three-level meta-analysis of seven studies (11 effect sizes) assessing motor symptoms-Overall Effect Size Results**


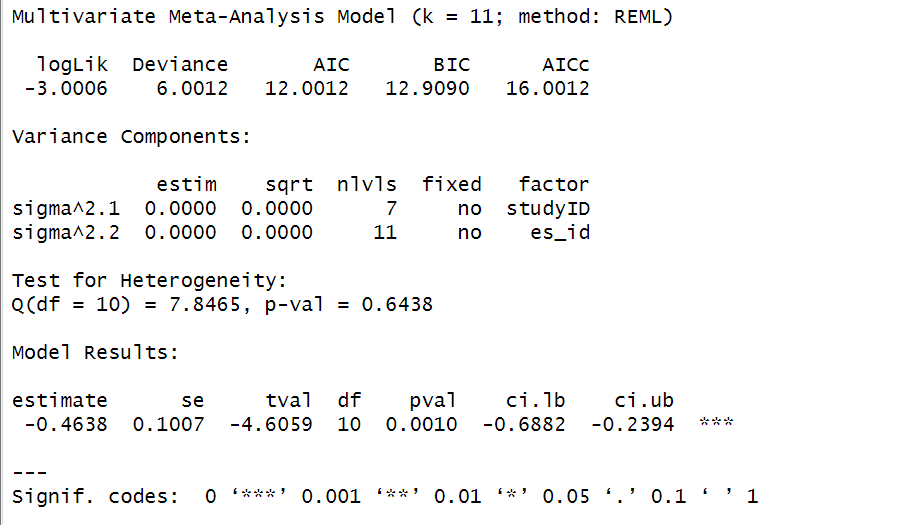


**Figure S35: The three-level meta-analysis of seven studies (11 effect sizes) assessing motor symptoms-Total variation**

**
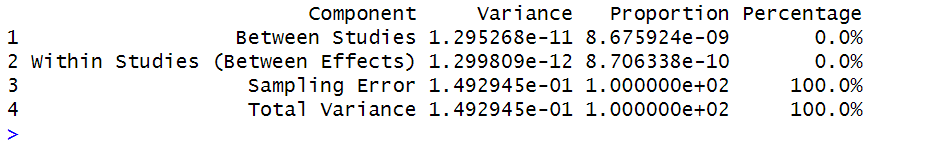
**

**Figure S36: The three-level meta-analysis of seven studies (11 effect sizes) assessing motor symptoms-*I^2^***


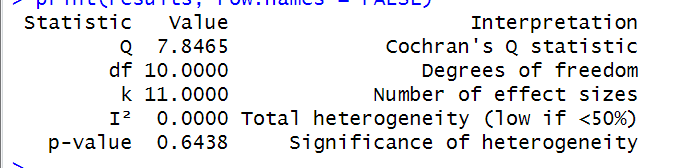


**Figure S36: The three-level meta-analysis of seven studies (11 effect sizes) assessing motor symptoms-Subgroup analysis(Intervention Type)**


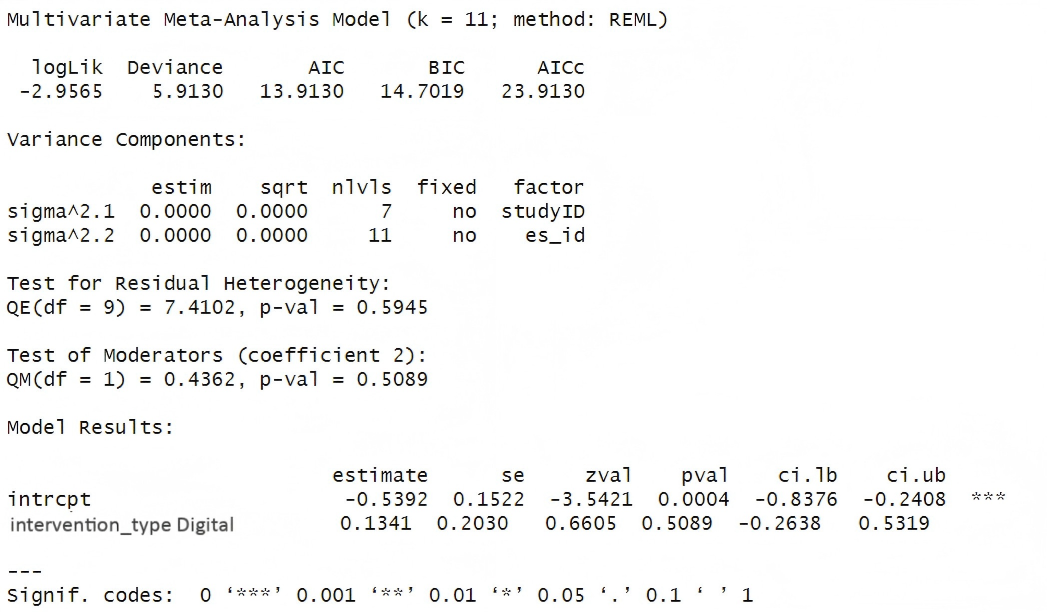


**Figure S37: The three-level meta-analysis of seven studies (11 effect sizes) assessing motor symptoms-Subgroup analysis(Follow-up duration_categorical)**

**
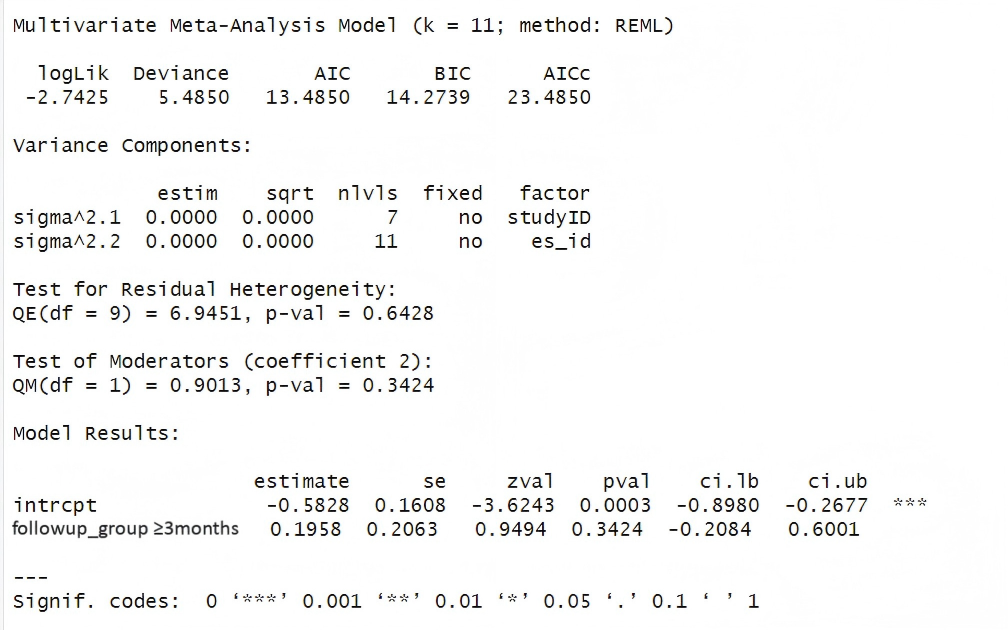
**

**Figure S38: The three-level meta-analysis of seven studies (11 effect sizes) assessing motor symptoms-Subgroup analysis(Follow-up duration_continuous)**


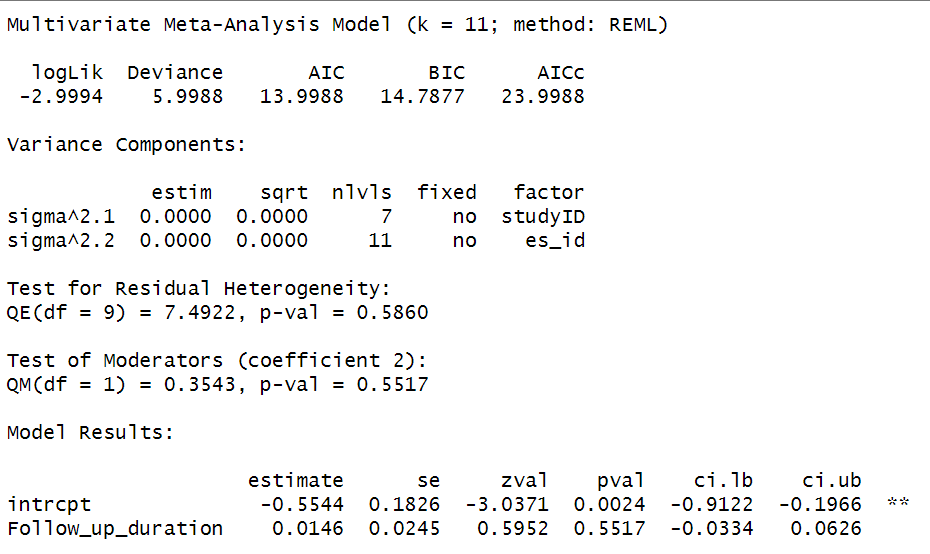


**Figure S39: The three-level meta-analysis of seven studies (11 effect sizes) assessing motor symptoms-Subgroup analysis(The relationship between effect size and follow-up time)**

**
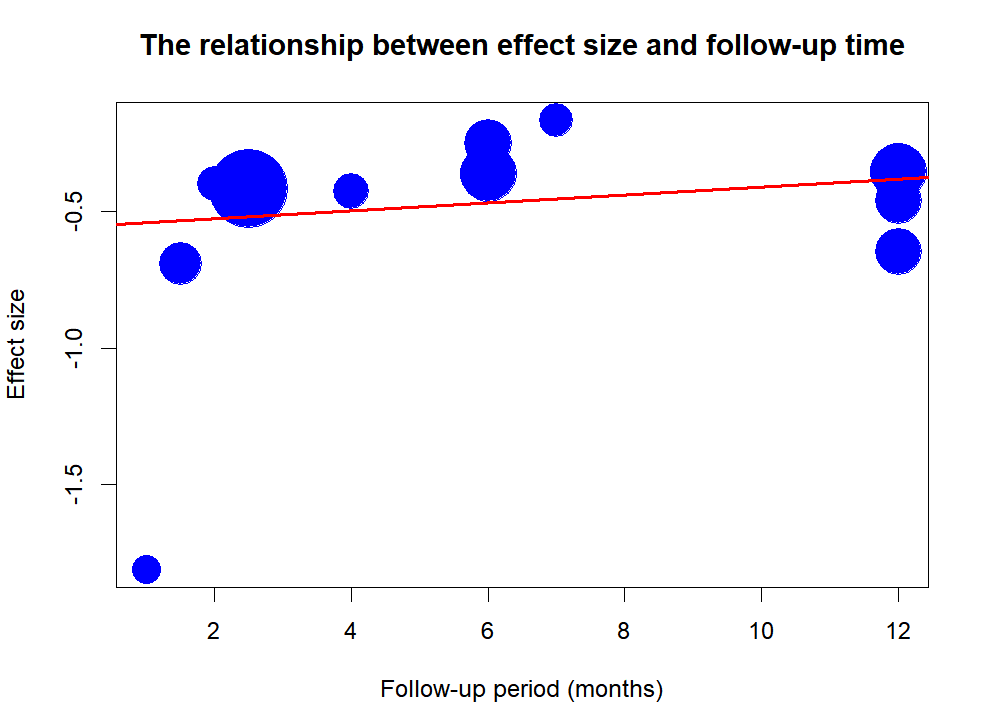
**

**Figure S40: The three-level meta-analysis of seven studies (11 effect sizes) assessing motor symptoms-Sensitivity analysis**


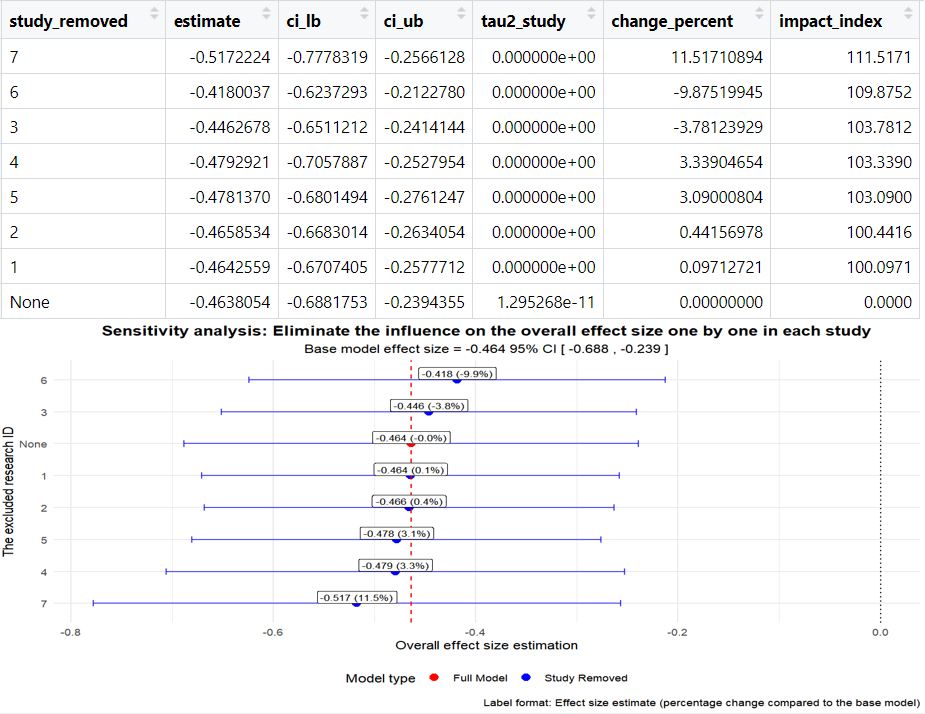


1=E Cubo 2017; 2=Rocio Del Pino 2023; 3=Sefa Eldemir 2023; 4=Michela GoffrEdo 2023; 5=Dustin A. Heldman 2017; 6=Pastana Ramos 2023; 7=Jayne R 2016

**Figure S41: The three-level meta-analysis of seven studies (11 effect sizes) assessing motor symptoms-Funnel plot and multilevel Egger's test**


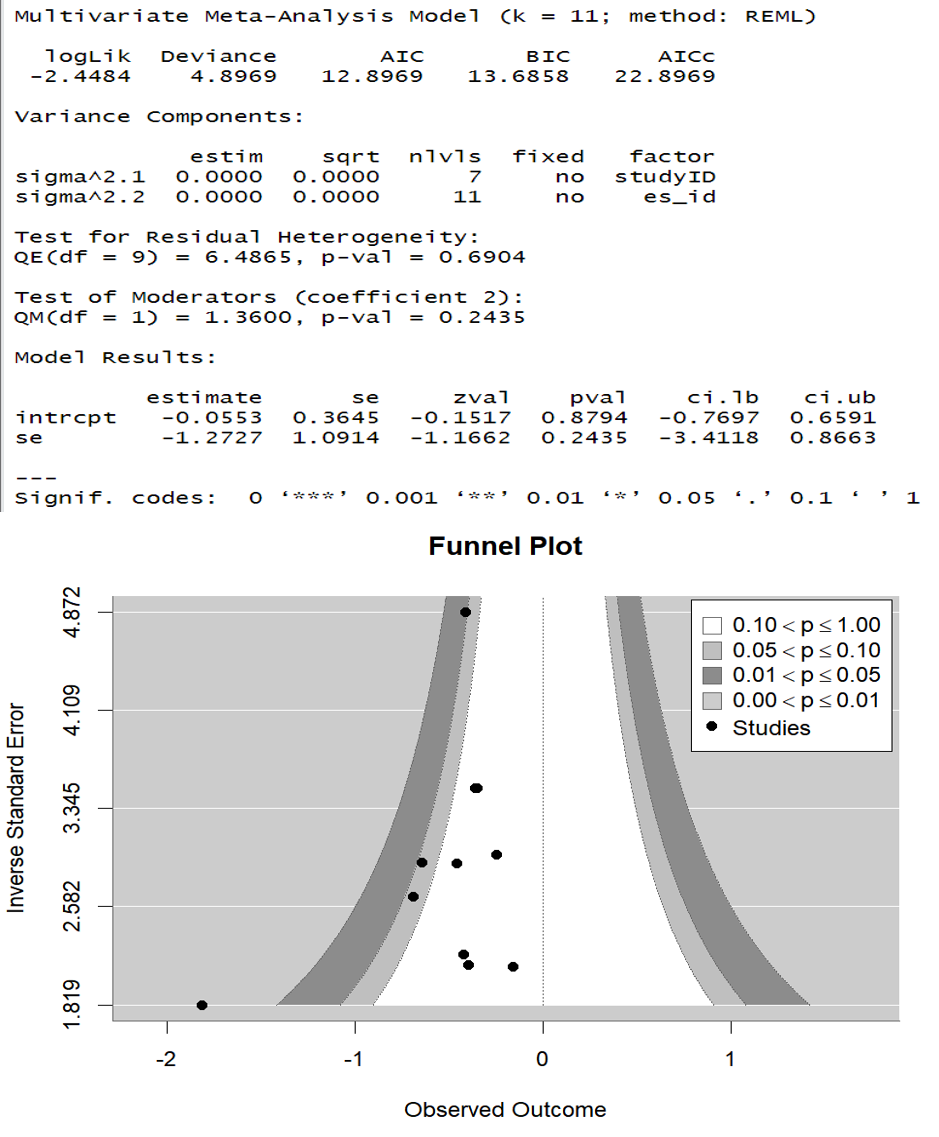


**Figure S42: Activities of daily living**


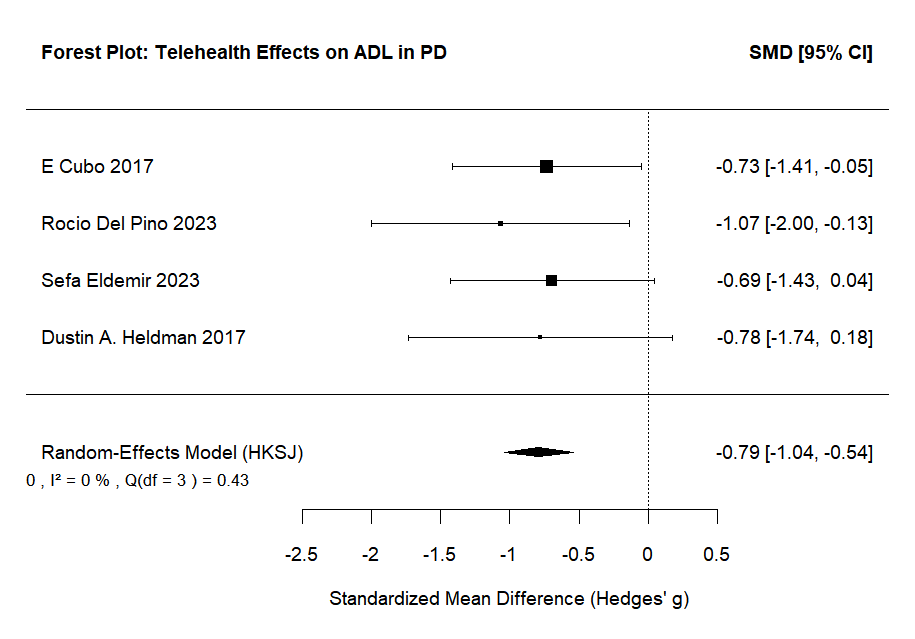


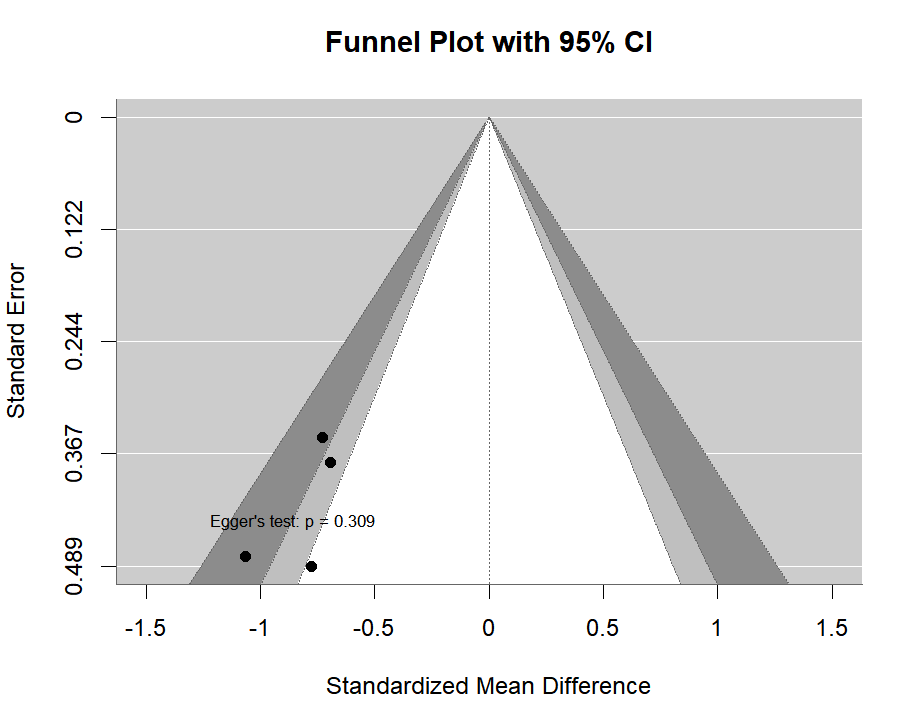


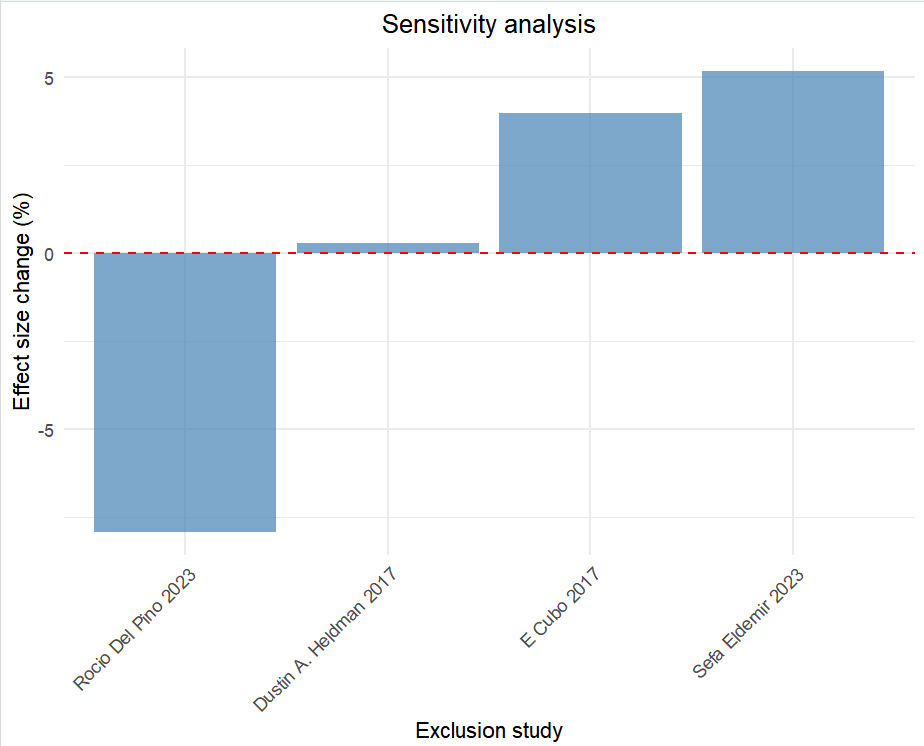


**Figure S43: The three-level meta-analysis of two studies (9 effect sizes) assessing cognition**

**-Overall Effect Size Results**


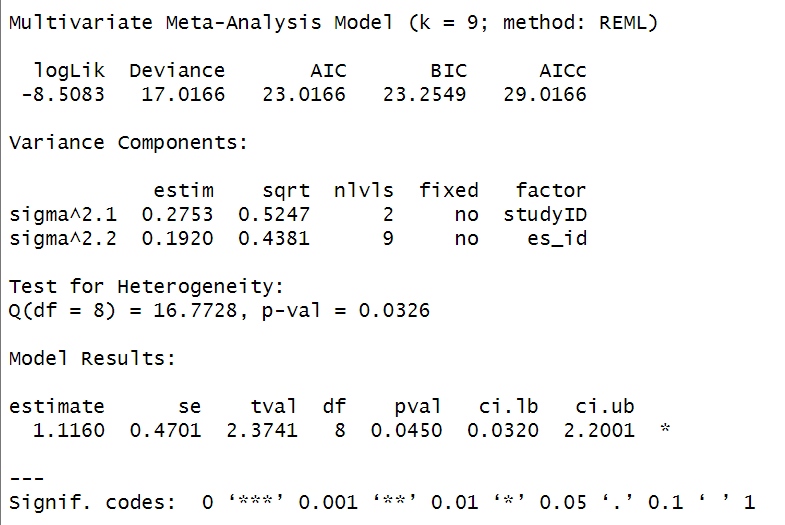


**Figure S44: The three-level meta-analysis of two studies (9 effect sizes) assessing cognition**

**-Total variation**

**
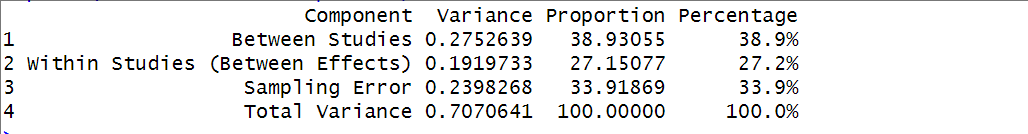
**

**Figure S45: The three-level meta-analysis of two studies (9 effect sizes) assessing cognition**

**-*I^2^***


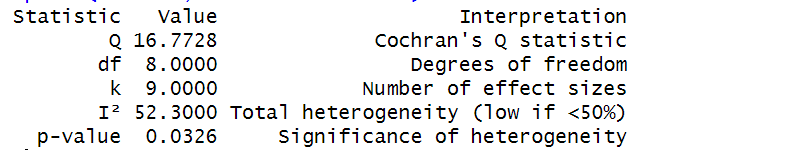


**Figure S46: The three-level meta-analysis of two studies (9 effect sizes) assessing cognition**

**-Subgroup analysis(Follow-up duration_categorical)**


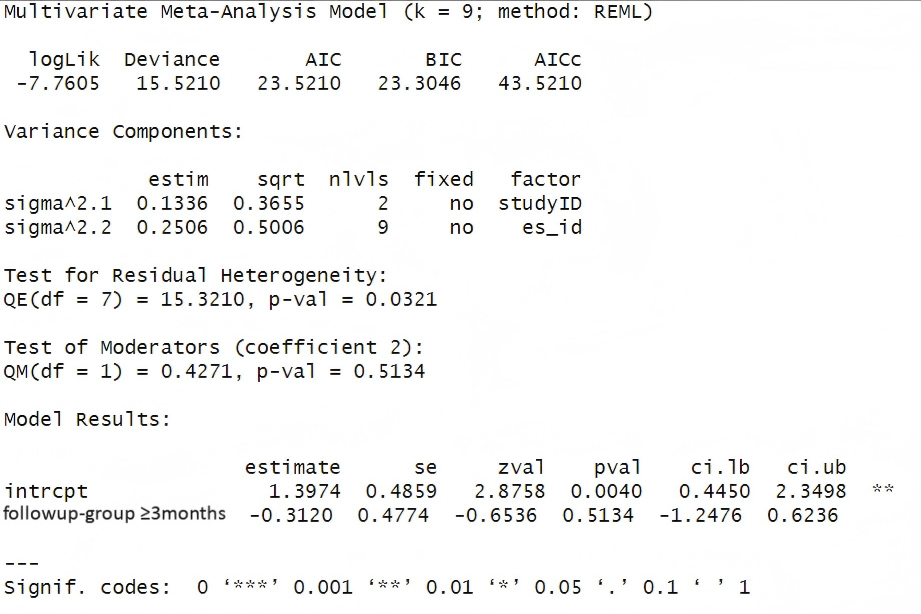


**Figure S47: The three-level meta-analysis of two studies (9 effect sizes) assessing cognition**

**-Subgroup analysis(Follow-up duration_continuous)**


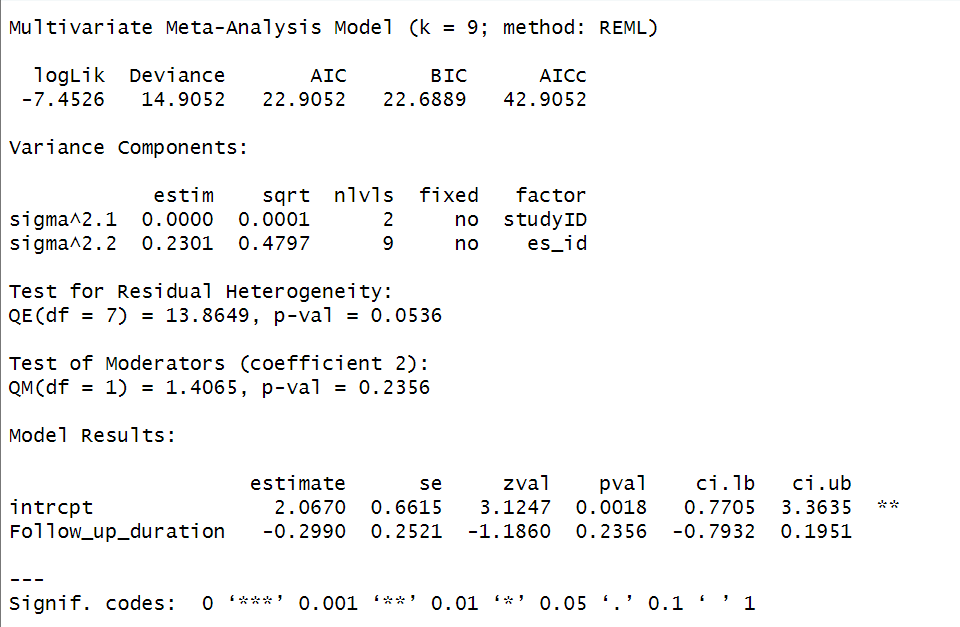


**Figure S48: The three-level meta-analysis of two studies (9 effect sizes) assessing cognition**

**-Subgroup analysis(The relationship between effect size and follow-up time)**


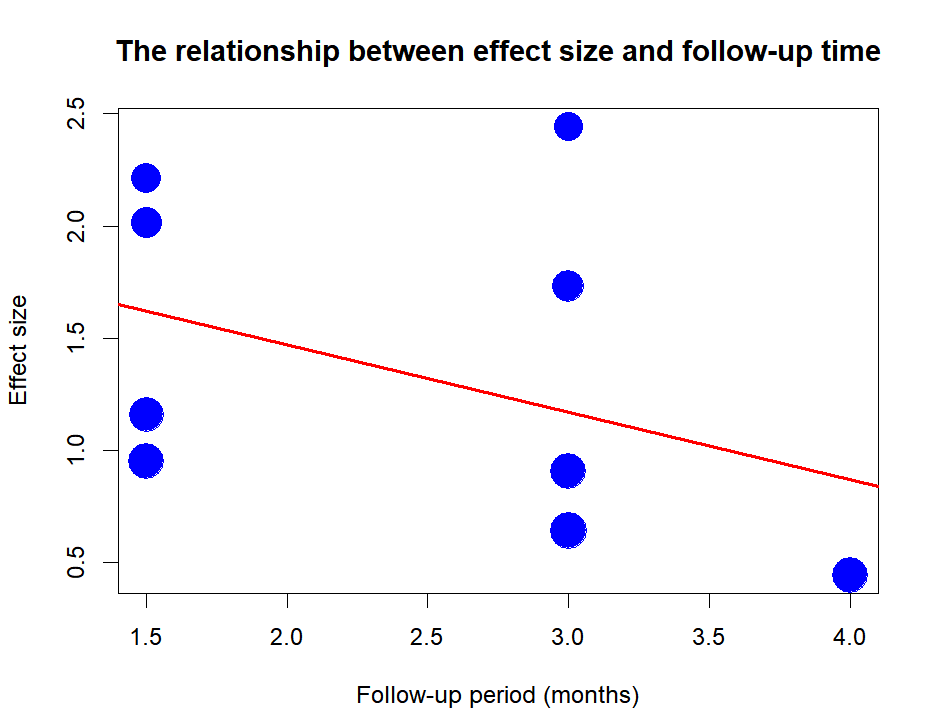


**Figure S49: The three-level meta-analysis of two studies (9 effect sizes) assessing cognition**

**-Funnel plot and multilevel Egger's test**


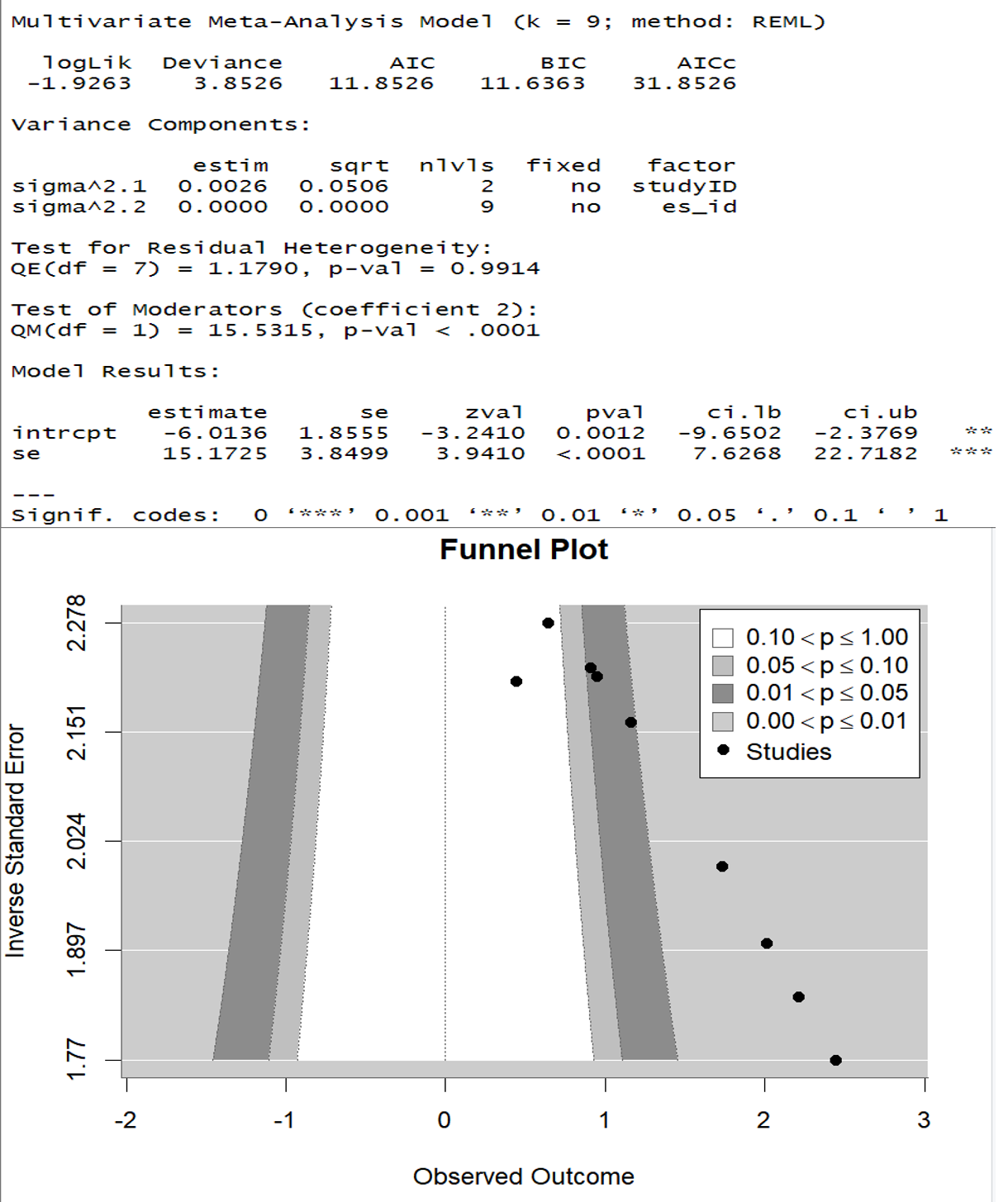

Supplement: Multimedia Appendix 4 [file mhealth_v14i1e70994_app4.doc]
